# The maize preligule band is subdivided into distinct domains with contrasting cellular properties prior to ligule outgrowth

**DOI:** 10.1242/dev.201608

**Published:** 2023-10-30

**Authors:** Wesley R. Neher, Carolyn G. Rasmussen, Siobhan A. Braybrook, Vladimir Lažetić, Claire E. Stowers, Paul T. Mooney, Anne W. Sylvester, Patricia S. Springer

**Affiliations:** ^1^ Department of Botany and Plant Sciences, Center for Plant Cell Biology, University of California, Riverside, CA 92521, USA; ^2^ Department of Molecular Biology, University of Wyoming, Laramie, WY 82071, USA; ^3^ Department of Molecular, Cell, and Developmental Biology, University of California, Los Angeles, CA 90095, USA; ^4^ California NanoSystems Institute, Los Angeles, CA 90095, USA

**Keywords:** Ligule, Maize, Auxin, Cell wall, AFM, Cell division, Boundary

## Abstract

The maize ligule is an epidermis-derived structure that arises from the preligule band (PLB) at a boundary between the blade and sheath. A hinge-like auricle also develops immediately distal to the ligule and contributes to blade angle. Here, we characterize the stages of PLB and early ligule development in terms of topography, cell area, division orientation, cell wall rigidity and auxin response dynamics. Differential thickening of epidermal cells and localized periclinal divisions contributed to the formation of a ridge within the PLB, which ultimately produces the ligule fringe. Patterns in cell wall rigidity were consistent with the subdivision of the PLB into two regions along a distinct line positioned at the nascent ridge. The proximal region produces the ligule, while the distal region contributes to one epidermal face of the auricles. Although the auxin transporter PIN1 accumulated in the PLB, observed differential auxin transcriptional response did not underlie the partitioning of the PLB. Our data demonstrate that two zones with contrasting cellular properties, the preligule and preauricle, are specified within the ligular region before ligule outgrowth.

## Teaser

**Summary:** Changes in cell geometry, division orientation and cell wall mechanics underlie maize ligule morphogenesis. The establishment of mechanically distinct epidermal domains coincides with topographical changes during early ligule outgrowth.

## INTRODUCTION

Organogenesis in plants is dependent on positionally determined cell patterning and regulation of cell division and expansion. Morphogenesis and differentiation ultimately give rise to diverse leaf shapes with distinct domains, such as petiolate leaves in many eudicots and sheathing leaves in grasses (Moon and Hake, 2011). Leaf morphogenesis involves the establishment of genetically defined developmental boundaries and accompanying shifts in cell, tissue and organ polarity. An emerging leaf acquires organ polarity in three dimensions relative to the plant axis, including proximodistal (apical to basal), mediolateral, and adaxial to abaxial (inner to outer leaf side). Changes in the rate and direction of cell division and expansion in these three polar dimensions are key components of organogenesis and contribute to sculpting leaf shape (Echevin et al., 2019). How cell division and cell expansion contribute to establishing boundaries remains an open question and is crucial to understand how leaf domains develop.

Boundary domains are often established before morphogenesis and contain distinct cells with altered signaling and cell wall properties. A well-studied boundary in plants is at the shoot apical meristem (SAM), where a leaf emerges and acquires new organ polarity. Cell growth is repressed at the SAM-leaf boundary, thereby facilitating the separation and emergence of the incipient leaf from the meristem (Hussey, 1971; Kwiatkowska and Dumais, 2003). The boundary is maintained at the base of the leaf throughout development and can be recapitulated at other locations as leaf domains differentiate (Bouré et al., 2022; Johnston et al., 2014; Nahar et al., 2012; Xiao et al., 2022).

Mutations in *Arabidopsis* genes encoding boundary-defining transcription factors such as *CUP-SHAPED COTYLEDON2* (*CUC2*), *CUP-SHAPED COTYLEDON3* (*CUC3*), *LATERAL ORGAN BOUNDARIES* (*LOB*) and *LATERAL ORGAN FUSION1* (*LOF1*) lead to improper organ separation due in part to derepression of cell division and expansion within the boundary domain (Bell et al., 2012; Gendron et al., 2012; Hibara et al., 2006; Lee et al., 2009). Although mutant studies highlight the importance of boundary-defining transcription factors, the mechanisms regulating cell growth in boundaries are not fully understood. LOB regulates brassinosteroid (BR) catabolism in boundary domains as one mechanism of limiting growth (Arnaud and Laufs, 2013). Cell wall-modifying genes are enriched among the transcriptional targets of BRs, and BR signaling is known to affect cell wall composition and structure, suggesting that cell wall biophysical properties are a component of boundary function (Bai et al., 2012; Graeff et al., 2021; Sun et al., 2010). Consistent with this, cell wall-related gene ontology terms are also significantly enriched among the transcriptional targets of LOB, whereas CUC2 represses many genes associated with cell wall loosening (Bell et al., 2012; Bouré et al., 2022; Cucinotta et al., 2018). Other experiments show that cell wall-related genes are enriched among highly translated transcripts in the boundary (Tian et al., 2014). In addition, cells in boundary domains have more rigid cell walls, as measured with atomic force microscopy (AFM) (Bouré et al., 2022; Sampathkumar et al., 2019). Changes in cell wall composition or remodeling activity could contribute to the decreased rate of cell expansion in boundary domains, but more experiments are needed to determine how the boundary function modulates growth. Although the SAM-leaf primordium boundary has been relatively well-studied, less is known about how other developmental boundaries are specified in plants and contribute to organogenesis.

A challenge to analyzing the SAM-leaf boundary is its physical inaccessibility. The maize leaf provides a unique opportunity to study an accessible boundary at the ligular region, which plays an important role in the proximodistal patterning of the leaf. The two largest domains of grass leaves are the proximal sheath and the distal blade, separated by the ligular region (Fig. S1), where several specialized structures develop. A thin epidermis-derived structure called the ligule emerges at the boundary between blade and sheath and covers the gap between consecutive ensheathing leaves. Also at the blade-sheath junction, two wedge-shaped structures called auricles develop on both sides of the midrib. Auricles are thought to facilitate the outward bending of the blade to optimize photosynthetic light capture (Emerson, 1912). The ligule is derived from a distinct region of the adaxial epidermis called the preligule band (PLB), a narrow linear boundary domain between the preblade and presheath of the leaf primordium (Becraft et al., 1990; Sylvester et al., 1990). Owing to physical proximity and the genetic links between the PLB, ligule and auricle, the adaxial epidermal portion of the pre-auricle is also hypothesized to be derived from the PLB and/or from blade tissue adjacent to the upper boundary of the PLB. These hypotheses have not yet been resolved.

Transcription factors in maize that contribute to the development of the ligular region have been identified, including LIGULELESS1 (LG1) and LIGULELESS2 (LG2), which specify ligule and auricle development in a partially redundant manner (Becraft et al., 1990; Walsh et al., 1998). Single mutants *lg1-R* and *lg2-R* affect the position of the blade-sheath boundary and alter the pattern of ligule and auricle development, whereas the *lg1-R*; *lg2-R* double mutant has an indistinct blade-sheath boundary and lacks both ligule and auricle (Foster et al., 2004; Harper and Freeling, 1996). Mutations in *lg1* and *lg2* genes result in more vertical leaf angles because the auricles do not develop properly (Emerson, 1912). Rice *lg1* and *lg2* mutants display phenotypes similar to those in maize, suggesting functional conservation in grasses, despite differences in ligular region structures (Lee et al., 2007; Wang et al., 2021).

The ligule is a clear example of how cell division and expansion contribute to establishment of a boundary. Changes in division rate and orientation occur during the earliest stage of ligule morphogenesis (Becraft et al., 1990; Sharman, 1942; Sylvester et al., 1990). Cells divide more frequently in the adaxial epidermis in the PLB based on the emergence of new cross-walls (Freeling, 1992; Sylvester et al., 1990), and *LG1* transcript accumulates at this site of increased division (Johnston et al., 2014; Moreno et al., 1997). Several rounds of epidermal anticlinal divisions (perpendicular to the surface), along with decreased cell expansion, reduce cell surface area (Becraft et al., 1990; Sylvester et al., 1990). The PLB becomes visible as a narrow band of small cells aligned laterally across the adaxial epidermis at the boundary between the blade and sheath domains. After several rounds of anticlinal divisions, periclinal divisions (parallel to the surface) are observed in both the PLB and the underlying ground tissue, and a ridge forms within the PLB (Sylvester et al., 1990). The auricle differentiates between the ridge and the blade while the ligule develops as a fringe of cells growing up and out from the PLB ridge (Freeling, 1992; Sylvester et al., 1990).

The proximodistal transcriptomic profile of the ligular region has been analyzed with high spatial resolution by laser-capture microdissection followed by RNA-seq in wild-type B73 and *lg1-R* mutants (Johnston et al., 2014), demonstrating that genes involved in leaf initiation and patterning at the SAM are redeployed later during ligule development. Notably, transcript levels of *KNOTTED1-LIKE HOMEOBOX1* (*KNOX1*) class and other boundary-associated genes such as *CUP-SHAPED COTYLEDON2-like* (*CUC2-like*; *nactf119*) are significantly higher in the PLB (Johnston et al., 2014). *In situ* hybridization has shown that *CUC2-like* transcripts were detected throughout the PLB early in development, but later became restricted to the distal zone of the PLB, where a cleft will form as the ligule grows out. Xiao et al. (2022) further supported the link between *lg2* and boundary-associated gene expression in the context of bract suppression in the inflorescence. These patterns of gene expression support the idea that the PLB functions as a boundary domain. Although the SAM-leaf boundary is characterized by a low mitotic rate (Hussey, 1971), the PLB displays increased cell division relative to neighboring regions (Becraft et al., 1990; Sylvester et al., 1990). Reduced cell size is a shared feature between the PLB and SAM-leaf boundary (Becraft et al., 1990; Hussey, 1971).

PIN-FORMED (PIN) auxin efflux genes and several other auxin-responsive genes are upregulated in the PLB, suggesting a role for auxin in ligule development (Johnston et al., 2014; Moon et al., 2013). Polar auxin transport and high auxin transcriptional responses are associated with the initiation and development of many structures during plant development, including leaves, branches, lateral roots, root hairs and vasculature (Barazesh and McSteen, 2008; Bennett et al., 2014; Du and Scheres, 2018; Hajný et al., 2022; Jones et al., 2009; Leyser, 2018; McSteen and Leyser, 2005; Pitts et al., 1998; Scarpella et al., 2010). Transcriptomic experiments indicate that before and during ligule development, auxin responses are higher in the blade than in the sheath (Leiboff et al., 2020; Johnston et al., 2014). Auxin dynamics at the blade-sheath boundary are thought to be involved in PLB development and ligule outgrowth (Johnston et al., 2014). More recently, polar auxin transport was shown to be necessary for ligule development (Satterlee et al., 2023). One proposed model is that PIN-like, *KNOX1* and *CUC2-like* genes are expressed in the early PLB, but subsequent antagonism by auxin responses restricts the expression of boundary-associated genes to the cleft, resulting in further refinement of the PLB into subdomains (Johnston et al., 2014).

Here, we document the stages of ligule development and identify changes in cell wall rigidity in different regions. Ligule morphology correlates with sheath length and provides a convenient proxy for estimating ligule developmental stage. During early ligule outgrowth, we compared cell depth, division orientation and cell wall rigidity along the proximodistal axis. There was a clear divergence in cellular properties between proximal and distal PLB-derived domains before ligule outgrowth. Hypothesizing that auxin dynamics may underlie this differentiation, we examined the accumulation of auxin reporters during ligule development. Although the auxin transporter PIN1a marked with yellow fluorescent protein (YFP) was observed in the PLB, we did not detect local differences in auxin transcriptional responses before ligule outgrowth. Our findings of cell growth patterns and biophysically distinct regions within the PLB may be explained by structural remodeling of cells required for the establishment and physical separation of a new axis associated with ligule outgrowth.

## RESULTS

### Ligule developmental stages correlate with sheath length

**Fig. 1.**
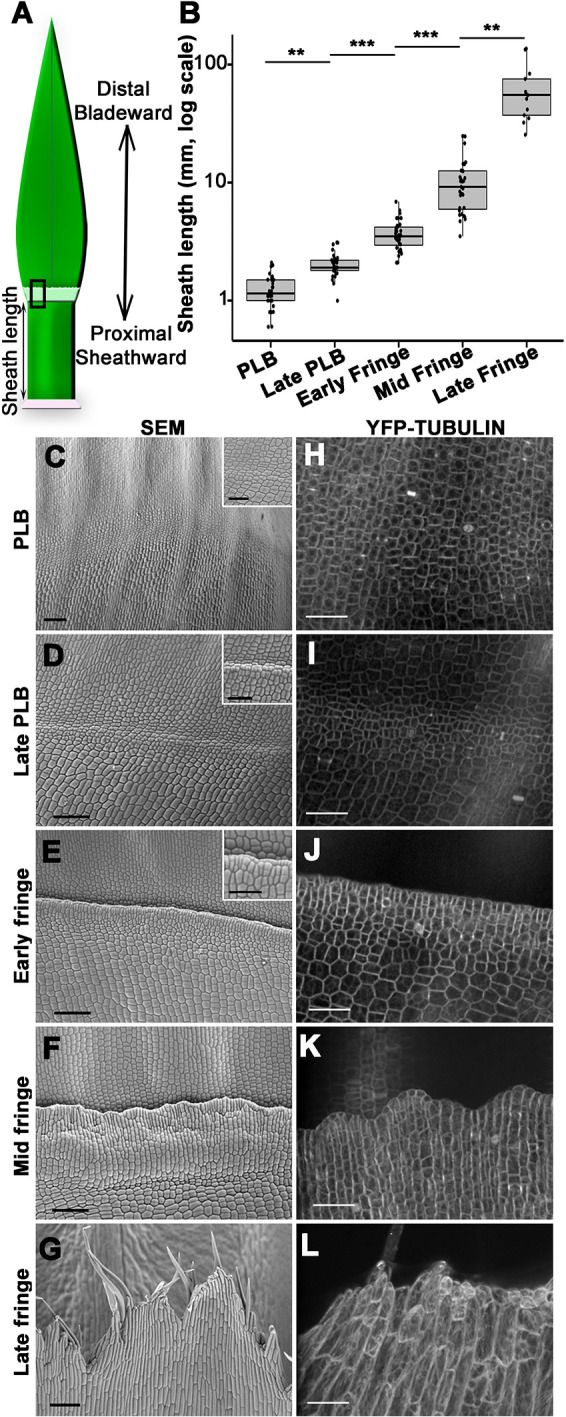
**Stages of ligule development correlate with sheath length.** (A) Cartoon outlining maize leaf domains and proximodistal axis. (B) Box and whisker plot showing that sheath height correlates with ligule stage. **P<0.05, ***P<0.01 (Kruskal-Wallis test with Dunn's post-hoc and Benjamini-Hochberg *P*-value adjustment for multiple comparisons). Box plot shows the first to third quartiles as boxes, with the center line indicating the median. The rest of the range, excluding outliers, is indicated by the whisker lines. All data points, including outliers, are shown as dots. (C-G) Scanning electron micrographs show stages of ligule development in four-week-old plants. Scale bars: 100 μm. Higher magnification insets in C-E show small cells in the preligule band. Scale bars: 50 μm. (H-L) Ligule stages visualized via confocal microscopy using YFP-TUBULIN marker. Mid and late fringe micrographs are maximum projections of *z*-stacks 3-5 µm in depth. Scale bars: 50 μm.

To establish developmental reference stages for ligule morphogenesis, we characterized features such as topography and cell size. Existing literature describes the stages of ligule development relative to plastochron number, a value indicating the relative age of a leaf. Plastochron number is difficult to determine because it requires either sectioning or dissection down to the meristem (Johnston et al., 2014; Sylvester et al., 1990). For accuracy, we used sheath length as a reliable and convenient proxy for predicting the stage of ligule development (Fig. 1A,B). Stages of ligule development were characterized in relation to sheath length in 2-, 3- and 4-week-old plants (Fig. 1; Fig. S2). Sheath lengths were measured in sequentially dissected leaves and ligule regions were observed by scanning electron microscopy (SEM; Fig. 1C-G). Ligule developmental stages were also visualized using confocal microscopy of leaves expressing YFP-TUBULIN (Fig. 1H-L). Although the ligule develops continuously and progressively, morphological features of the ligule correlated significantly with sheath length in expanding adult leaves (Fig. 1B).

The stages distinguishable by SEM were defined as PLB, late PLB, and early, mid and late fringe. At a median sheath length of 1.2 mm, comparable with late plastochron 6 and early plastochron 7 (Johnston et al., 2014), the PLB consisted of a band of small cells spanning ∼60-100 μm in proximal/distal length. At this stage, a slight ridge was often visible at the blade-sheath junction, with an inflection point at the PLB (Fig. 1C,H). At the late PLB stage, this ridge was pronounced, with the adjacent sheath surface elevated above the blade surface (Fig. 1D,I). Median sheath length was 1.9 mm, comparable with late plastochron 7 (Johnston et al., 2014), and cell area reached a minimum at this stage (Table S1). Leaves in the early fringe stage had a median sheath length of 3.5 mm, comparable with plastochron 8. Ligule cells were aligned at the leading edge of the ridge, beginning to grow over the more distal PLB-derived cells (Fig. 1E,J). Cell area increased throughout the development of the fringe (Table S1). Leaves in the mid fringe stage had a median sheath length of 8.7 mm. The ligule appeared to be ‘corrugated’ and uneven relative to the plane of the leaf (Fig. 1F,K). At a median sheath length of 54.1 mm, the ligule was in the late fringe stage defined by elongate hair-like cells at the leading edge of the ligule, which projected over the developing auricle and blade (Fig. 1G,L). These observations demonstrate that ligule development correlates with sheath growth (Fig. 1B; Fig. S2), indicating that sheath length can be used to approximate the developmental stage of the ligule.

### Changes in cell division orientation and expansion are associated with PLB and fringe growth

**Fig. 2.**
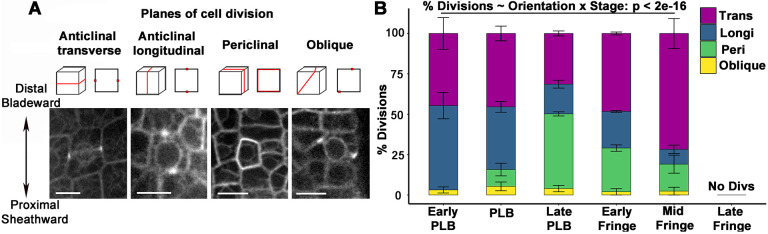
**Dynamic changes in division plane orientation during ligule development.** (A) Cartoons (top) show different division plane orientations as seen in 3D and in a single *z*-slice, along with examples of preprophase bands in cells expressing YFP-TUBULIN (bottom). Scale bars: 15 μm. (B) The percentage of dividing cells that exhibit each division orientation at each stage of ligule development. Error bars indicate standard error. *n*=3-5 leaves per stage, 11-46 mitotic cells per leaf. Significance determined via ANOVA.

Changes in cell division and expansion patterns are characteristic of the PLB. We used a live cell marker for microtubules, YFP-TUBULIN (Mohanty et al., 2009), to assess cell area and division plane orientation at each of the defined ligule stages. We calculated the relative frequencies of different divisions by classifying the orientation of preprophase bands, mitotic spindles and phragmoplasts (Fig. 2A).

Visualizing microtubule mitotic structures enabled us to discern an earlier developmental stage than was visible with SEM, which primarily identifies new cross walls as indicators of recent cell divisions (Sylvester et al., 1990). This early PLB stage was observed at sheath lengths of 0.3-1.1 mm, comparable with early plastochron 6 (Johnston et al., 2014). The predominance of longitudinal anticlinal divisions (>50%) at the blade-sheath junction was the distinguishing feature, the leaf surface was flat in the proximodistal direction and average cell area was ∼159 µm^2^ (Fig. 2B; Table S1). In the PLB stage, transverse anticlinal divisions were the most frequent and a low frequency of periclinal divisions was observed (Fig. 2B). In addition, the average PLB cell area decreased to ∼135 μm^2^ (Table S1). In the late PLB stage, periclinal divisions were observed most frequently (∼46%) and the average cell area was further reduced to ∼106 μm^2^. These results show reduced cell sizes in the PLB and shifts in division orientation from anticlinal to periclinal by the late PLB stage.

During the early fringe stage, periclinal divisions were reduced, with ∼48% of the divisions oriented in the transverse anticlinal plane. Cell expansion increased so that early fringe cells were ∼80% larger than late PLB cells. Mid fringe cells divided mostly in the transverse anticlinal orientation (71%), and cell area increased dramatically. Ligule cells in the late fringe stage no longer divided, but continued expanding, producing larger and more variably sized cells (Fig. 2B; Table S1). These results show that changes in division plane orientation contribute to early ligule emergence, with cell expansion driving ligule elongation at the late fringe stage.

**Fig. 3.**
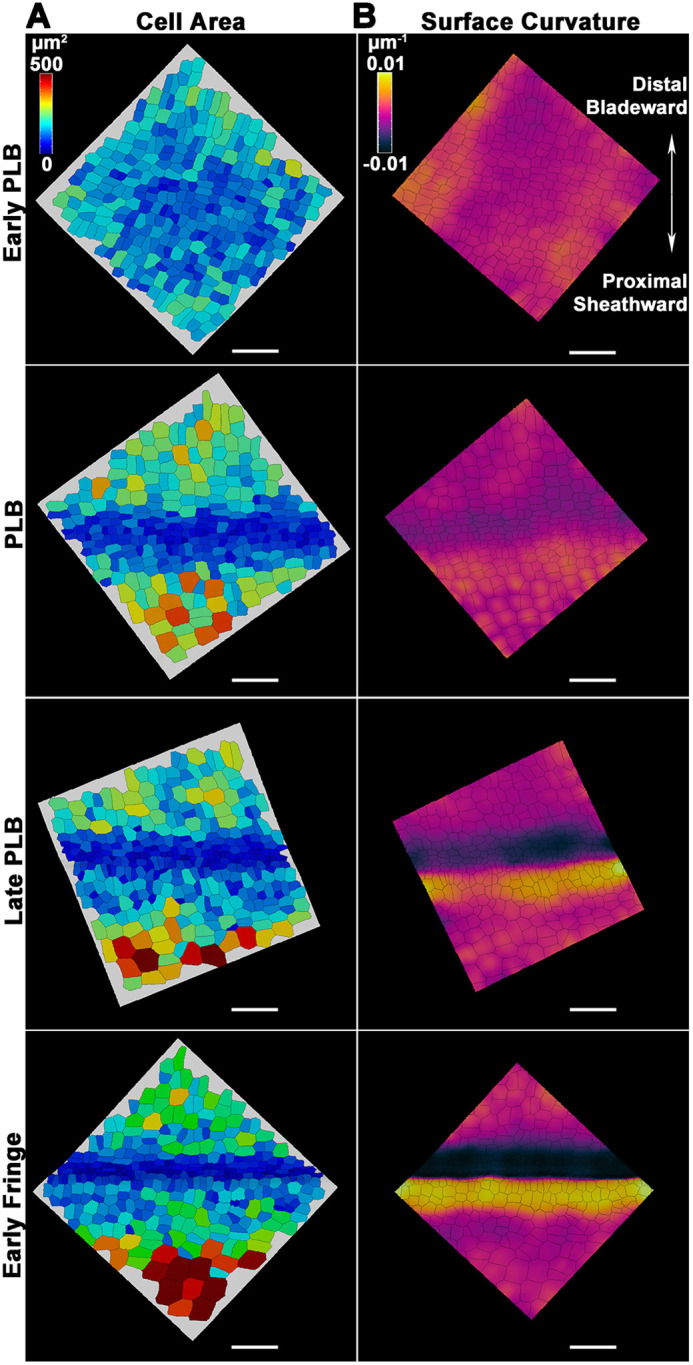
**Changes in cell area and surface topography during early ligule development.** Representative *z*-stacks from the ligular region were processed using MorphoGraphX. (A) Heatmaps showing spatiotemporal differences in cell area in the ligular region. (B) Same surfaces as in A, with heatmaps of average curvature of the surface at a neighborhood of 50 µm. Scale bars: 50 µm.

Representative confocal *z*-stacks were processed using the MorphoGraphX software package to better visualize changes in cell area and surface topography (Fig. 3; MorphoGraphX.org). The resulting projections showed a reduction in cell area during PLB development, although cell size in the ligular region was not uniform (Fig. 3A). Projections of average curvature showed the formation of a sharp ridge by the late PLB stage and revealed that the cells proximal to the ridge were noticeably larger than cells distal to the ridge (Fig. 3B).

### Differential cell expansion and division orientation within the PLB contribute to the formation of the preligule ridge

**Fig. 4.**
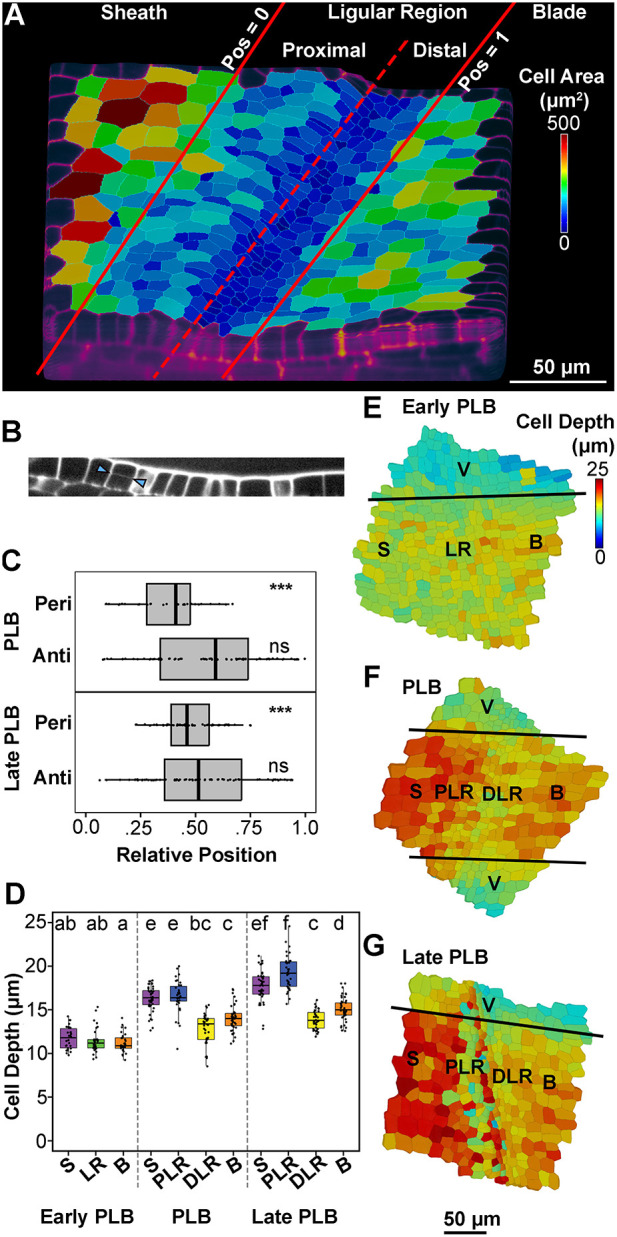
**Differential cell thickening and periclinal divisions contribute to formation of the preligule ridge.** (A) MorphoGraphX projection outlining the zones of the ligular region. Position 0 is the proximal extremity of the ligular region and position 1 is the distal extremity. (B) An orthoslice highlighting a periclinal division in the proximal ligular region (arrowheads). (C) Relative position of periclinal divisions within the ligular region was determined from confocal micrographs of plants expressing either CFP-Tubulin or TAN-YFP. ***P<0.01 (one-sample chi-squared tests for variance). ns, not significant (*P*>0.05). (D) Cell depth was measured from confocal *z*-stacks at anticlinal faces of cells that had not yet undergone periclinal divisions, in the ligular region of the maize leaf adaxial epidermis (*n*=3 leaves per stage, 10-15 cells per region per leaf). Significance was determined via Kruskal–Wallis test with Dunn's post-hoc, adjusting *P*-values via the Benjamini-Hochberg method for multiple comparisons, at an alpha of 0.05. Letter rankings indicate comparisons between all stages and epidermal zones: lowercase letters are used to label means, such that bars bearing different letters are statistically different from one another with a minimum *P*-value of <0.05. Box plots show the first to third quartiles as boxes, with the center line indicating the median. The rest of the range, excluding outliers, is indicated by the whisker lines. All data points, including outliers, are shown as dots. (E-G) 3D MorphoGraphX heatmaps of cell depth during early ligule development. The original scans for E and G are the same as those used for the corresponding stage in Fig. 3. B, blade; DLR, distal ligular region; LR, ligular region; PLR, proximal ligular region; S, sheath; V, vasculature.

During early ligule development, a ridge forms so that the sheath surface is elevated relative to the blade surface. Whereas periclinal divisions are known to contribute to the formation of this ridge, it is not clear whether they occur throughout the whole ligular region or are specific to a subset of cells that form the ligule. The relative positions of periclinal and anticlinal divisions within the ligular region were determined using either YFP-TUBULIN or TANGLED-YFP, a protein that localizes to the division site (Martinez et al., 2017; Walker et al., 2007; Movie 1). The ligular region was defined as the zone of reduced cell area between the blade and sheath (Fig. 4A). In the PLB stage, sporadic periclinal divisions were visualized in the proximal two-thirds of the ligular region but not in the distal one-third (Fig. 4B,C). In the late PLB stage, periclinal divisions were exclusively observed in the median 50% of the ligular region, localized to the nascent preligule ridge, but absent from both extremities (Fig. 4C). At both stages, anticlinal divisions were broadly distributed over the entire proximodistal length of the ligular region (Fig. 4C). Therefore, epidermal periclinal divisions occur in the proximal ligular region, but not in the distal cells that contribute to the auricle.

Although periclinal divisions in the PLB and underlying mesophyll cells are known to contribute to the formation of the ridge at the blade-sheath boundary (Sharman, 1941), proximodistal differences in cell thickness (depth) have not been quantified. To determine whether differential thickening of epidermal cells contributed to the formation of the ridge, we measured cell depth in the epidermis of the sheath, ligular region and blade during the early, mid and late stages of PLB development (Fig. 4D). In the early PLB stage, cell depth was uniform, averaging 11-12 μm in the sheath, PLB and blade (Fig. 4D). During the PLB stage, the sheath and proximal ligular region cells averaged 16.4 μm deep, whereas the distal ligular region and blade cells were 13 μm deep (Fig. 4D). This relative thickening of the sheath coincided with the formation of the ridge. By the late PLB stage, the rate of periclinal divisions in the PLB increased and the preligule ridge became more pronounced, with cells on the proximal side of the ridge averaging 19.2 μm deep (Fig. 4D). Meanwhile, the distal PLB-derived cells were the thinnest in the epidermis, averaging 13.9 μm deep. We used MorphoGraphX to extract cell depth data from representative confocal *z*-stacks, which largely agreed with our measurements (Fig. 4E-G). Our findings regarding epidermal cell depth are consistent with previously published transmission electron microscopy images of the developing ligule (Sharman, 1941, 1942). These data show that differential cell thickening contributes to the changes in epidermal topography during the early stages of ligule development.

### Mechanical changes within the epidermis precede ligule outgrowth

Differences in cell size and division orientation indicated that two zones with contrasting cellular behavior are established in the ligular region before emergence of the fringe. The elastic properties of the cell wall often correlate with cell expansion and reflect physical differences both between different cell populations and between subcellular cell wall domains (Bou Daher et al., 2018; Peaucelle et al., 2011). We sought to identify cell wall mechanical patterns in epidermal cells during development of the ligular region. AFM uses a physical probe to measure the topography and various physical characteristics of surfaces. AFM data were used to calculate indentation modulus (IM), which is the complex elastic stiffness of the area being indented. High IM values indicate greater rigidity.

**Fig. 5.**
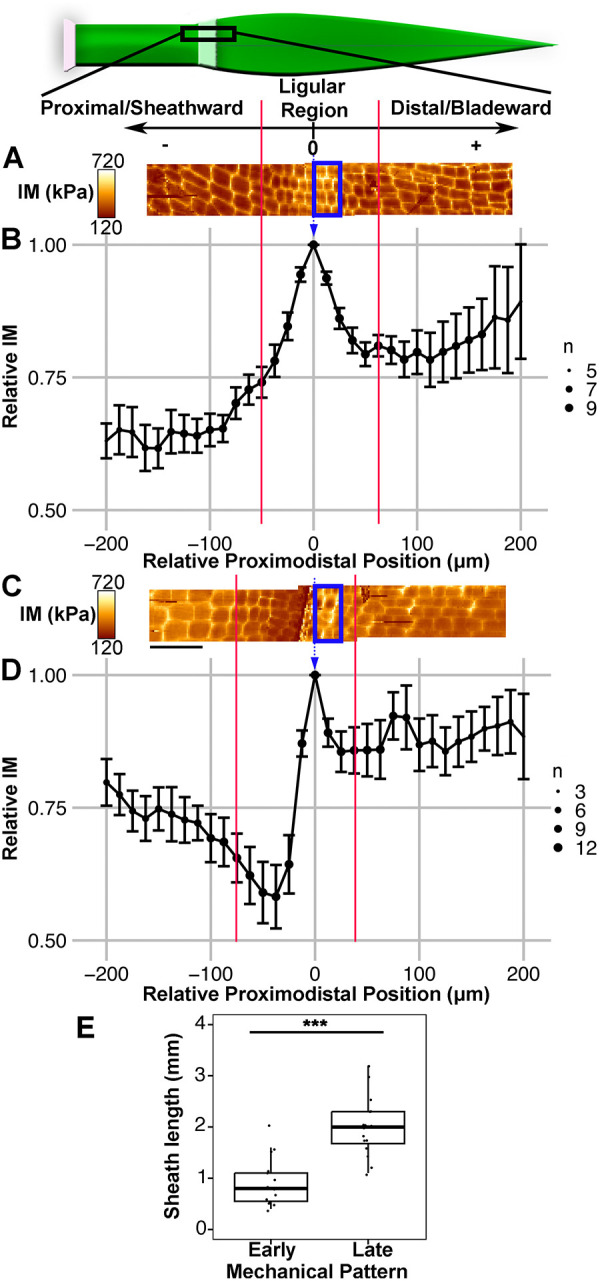
**AFM analysis of ligular region reveals two distinct mechanical phases during ligule development.** Scale and cartoon at top show orientation of leaf and region of interest for AFM scans and sliding window analysis, with the local maximum in IM for each leaf set as position 0. (A) Representative scans of leaf in the PLB stage reveals a local maximum in IM within the PLB. Two overlapping 50×200 μm scans are shown. Blue box indicates the position of the window for the measurement at relative position 0. Red lines indicate limits of the ligular region. (B) Sliding window analysis averaging all B73 samples exhibiting the early pattern (early PLB and PLB stages; *n*=9). (C) Representative scans of leaf in the early fringe stage. (D) Sliding window analysis averaging all B73 samples exhibiting the late pattern (late PLB and early fringe stages, *n*=13). A and C are to scale relative to B and D. Scale bars: 50 μm. Size of dot (*n*) indicates coverage at that relative position. Error bars indicate standard error. (E) Sheath lengths of leaves with the early and late mechanical patterns. ***P<0.01 (Mann–Whitney *U*-test). Box plot shows the first to third quartiles as boxes, with the center line indicating the median. The rest of the range, excluding outliers, is indicated by the whisker lines. All data points, including outliers, are shown as dots.

We used AFM to measure the rigidity of cell walls across epidermal regions in B73 leaves from the early PLB stage through the early fringe stage. Periclinal walls had relatively low rigidity, whereas anticlinal walls had higher rigidity, consistent with previous experiments in plasmolyzed tissue (Bou Daher et al., 2018; Peaucelle et al., 2011; Sampathkumar et al., 2019). To reveal tissue-scale patterns in rigidity along the proximodistal axis, we analyzed the AFM scans using a sliding window approach (see Materials and Methods; Fig. 5). Generally, the sheath had lower average rigidity than the blade at all stages of development. During early PLB and PLB stages, the central ligular region was the most rigid epidermal zone (Fig. 5A,B). During late PLB and early fringe stages, a different mechanical pattern was observed in the ligular region. A distinct ‘transition’ zone at the proximal end of the ligular region contained cells that were similar in shape to the sheath cells, but smaller and mechanically softer. The center of the ligular region had small cells with the lowest average rigidity. The distal ligular region, meanwhile, remained the most rigid epidermal zone (Fig. 5C,D). Samples exhibiting the ‘late’ mechanical pattern had significantly higher sheath lengths than those exhibiting the ‘early’ mechanical pattern, confirming that the two patterns occur at distinct developmental stages (Fig. 5E). Similar results were obtained in Mo17 leaves (Fig. S3), indicating that these patterns were not unique to the B73 genetic background. Therefore, a change in mechanical properties occurs between the PLB and late PLB stages, with significant softening in the middle of the PLB, while the distal pre-auricle region remains rigid.

To determine when the mechanical changes in the ligular region occur relative to ligule outgrowth, we directly compared topographical features to the rigidity data from the same AFM scans. In PLB-stage leaves, a shallow ridge was visible in the ligular region (Fig. S4A,B). Late PLB stage leaves had a steeper ridge, but relatively flat blade and sheath regions (Fig. S4C,D). The ligule grows out from this ridge, extending over the pre-auricle cells toward the blade. At early stages, the most rigid cells were centrally located in the PLB, but at later stages were located more distally, consistent with the position of the nascent ligule-auricle cleft. A distinct low-rigidity band, located on the crest of the ridge, was observed in the late PLB and early fringe stages (Fig. 5D; Fig. S4C,D). Cell wall softening in the late PLB stage correlates with the increases in cell area during the early fringe stage.

The sliding window method revealed two distinct mechanical patterns in the epidermis; however, this analysis may be biased because anticlinal walls are perceived as more rigid than periclinal walls in plasmolyzed tissue (Peaucelle et al., 2011) and cell size varies between epidermal regions. To avoid potential measurement bias due to cell size differences, we manually resampled rigidity from the AFM scans to compare transverse, longitudinal and periclinal cell wall segments (Fig. S5; Table S2). Generally, manual resampling confirmed the trends reported above, producing rigidity profiles that were similar to the sliding window analyses (Fig. 5; Fig. S5A,B; Table S2). In addition, considering each wall orientation separately allowed us to assess elastic asymmetry, defined as differences in the rigidity of different wall orientations (Bou Daher et al., 2018; Fig. S5C,D; Table S3). For example, in early stage leaves the average rigidity of transverse walls was higher than that of longitudinal walls in the blade and sheath, but not in the PLB, indicating reduced elastic asymmetry in the PLB cells (Fig. S5C; Table S3). The softer longitudinal walls in the blade and sheath are consistent with the primary direction of organ growth. Lastly, this approach enabled us to compare the average rigidity between the early and late stages (Table S4), revealing that the sheath anticlinal walls rigidified significantly in the late stage while the cells on the preligule ridge softened. Manual resampling supported the tissue-level patterns in rigidity observed with the sliding window approach and enabled further comparisons between the two mechanical stages and cell wall segments with different orientations.

### The pattern of PIN1a-YFP signal changes during ligule development and is ubiquitous in ligule cells

**Fig. 6.**
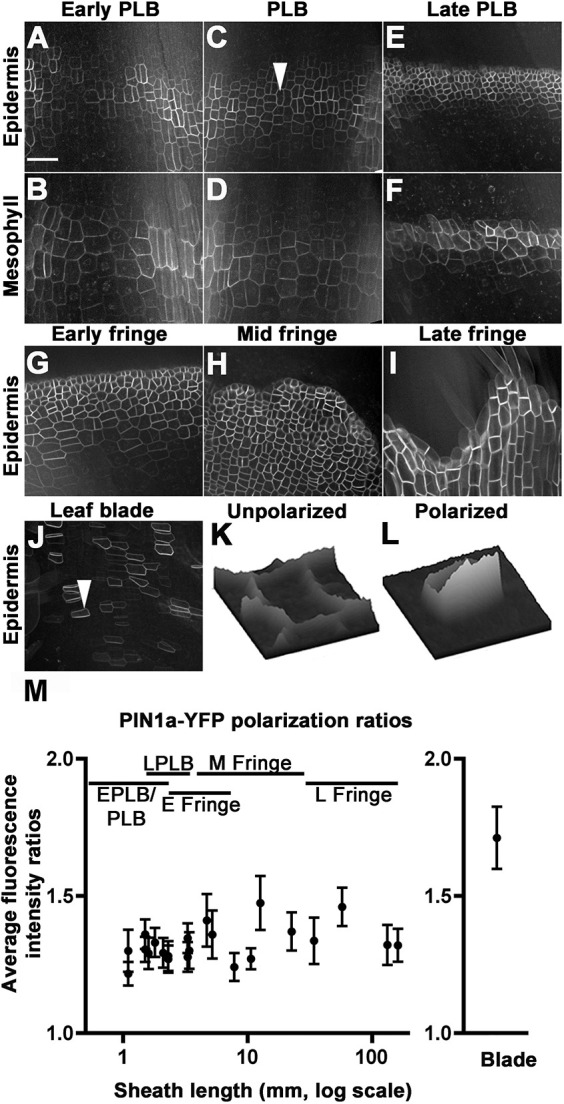
**PIN1a-YFP localization in the PLB and ligule.** (A-F) Panels A,C,E are single *z*-plane images of epidermal cells in early PLB, PLB and late PLB, respectively; B,D,F are corresponding single *z*-planes of the next cell layer in the subtending mesophyll. (G-I) PIN1a-YFP was observed in the early fringe (G), mid fringe (H) and late fringe (I). (J) PIN1-YFP signal was peripheral in blade cells above the ligule fringe. (K,L) Relative fluorescence intensity surface plots are displayed for the cell indicated by arrowheads in (C), showing a PLB cell with low polarity, and in (J), showing a blade cell with increased polarity. (M) PIN1a-YFP polarization ratios compared at ligule stages and in the blade, as calculated from 30 or more cells from four different plants. Error bars are 95% confidence intervals. Blade cells were significantly more polarized than PLB or fringe cells (*P*-value≤0.01 using the Kolmogorov-Smirnov test). Scale bar: 50 μm.

**Fig. 7.**
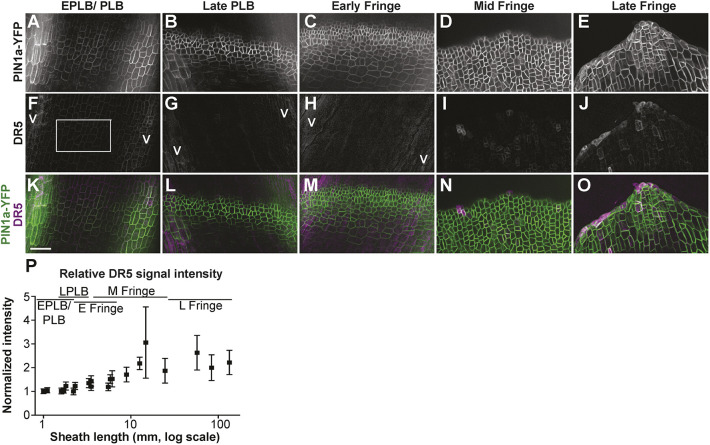
**PIN1a-YFP and DR5rev:mRFPer (DR5) localization during ligule development.** (A-O) PIN1a-YFP accumulation is shown in the top panels (A-E); DR5 in the middle panel (F-J), with merged PIN1a-YFP (green) and DR5 (magenta) in the bottom panel (K-O). (P) Normalized DR5 fluorescence intensity values. Each point is the average of three or more measurements per sample. DR5 intensity values increased significantly and were more variable during later ligule stages (F-test, *P*<0.01). Error bars show standard error. Scale bar: 50 μm.

Auxin has roles in many aspects of leaf development, including specification of founder cells (Reinhardt et al., 2003; Scanlon, 2003). In addition, application of exogenous auxin was sufficient to induce cell wall biochemical and mechanical changes in the SAM (Braybrook and Peaucelle, 2013). Previous work has shown that PIN auxin-efflux carrier transcripts *PIN1a*, *PIN1c* (*pin3*), *PIN5* and *SoPIN1* (*PIN1d* or *PIN4*) accumulate in the PLB, and PIN1a-YFP signal is strong in the PLB and underlying mesophyll (Conklin et al., 2019; Johnston et al., 2014; Moon et al., 2013), suggesting a role for auxin in ligule specification and/or outgrowth (Johnston et al., 2014). Live cell imaging of PIN1a-YFP was conducted during all stages of ligule development. At the early PLB stage, PIN1a-YFP localized to the plasma membrane of cells over the vasculature in both the epidermis and mesophyll (Fig. 6A,B). In the PLB stage, PIN1a-YFP was observed uniformly in the PLB and underlying mesophyll (Fig. 6C,D). The PIN1a-YFP-expressing zone consistently narrowed from ∼60 μm at the PLB stage to ∼45 μm at the late PLB stage, with signal only in the small PLB cells and underlying mesophyll, and not in the cells at the extremities of the ligular region (Fig. 6E,F). In the early fringe stage, the PIN1a-YFP-accumulating zone expanded only in the sheathward/proximal direction (Figs 6G, 7C). PIN1a-YFP signal was observed in ligule cells at all stages of fringe development (Fig. 6G-I). The narrowing of the zone containing PIN1a-YFP signal correlates with the increase in the periclinal division rate in the late PLB stage and precedes the outgrowth of the ligule fringe.

### PIN1a-YFP is less polarized in the PLB and ligule fringe compared with the blade

PIN proteins are auxin efflux transporters, and polar localization of PINs can result in directional auxin flow. PIN1 polarization, defined as asymmetric accumulation of the protein between polar domains of the plasma membrane, correlates with the direction of auxin transport (Wiśniewska et al., 2006). We examined PIN1a-YFP localization in the developing ligule, as an indication of directional auxin transport. We compared the relative polarity of PIN1a-YFP in developing ligule cells to that of blade epidermal cells (Fig. 6K-M). In contrast to the blade, where clear PIN1a-YFP polarity was observed (Fig. 6J), PIN1a-YFP localization in PLB and ligule cells appeared to be relatively nonpolar. Consistent with this observation, the fluorescence intensity ratio of PIN1a-YFP in the blade was 1.71±0.11, showing that the PIN1a-YFP is polarized in the blade, as expected (Fig. 6L,M). PIN1a-YFP was primarily localized to the rootward side of blade epidermal cells, consistent with basipetal auxin transport in leaf primordia (Sawchuk et al., 2013; Scarpella et al., 2010). In contrast, PIN1a-YFP signal was significantly less polarized in the PLB and the forming ligule (Fig. 6K,M), with mean ratios ranging from 1.21±0.04 to 1.47±0.09 (Fig. 6M). Whereas PIN1a-YFP signal was strong in the PLB and ligule throughout development, its subcellular localization was relatively nonpolar.

### Auxin transcriptional responses reported by DR5 are low in the PLB and increase during ligule elongation

*DR5* is a synthetic promoter containing auxin response elements, which can be used in combination with reporters to approximate auxin transcriptional responses (Ulmasov et al., 1997). We examined expression of *DR5rev:mRFPer* (*DR5*) in plants coexpressing *PIN1a-YFP* (Fig. 7) to determine whether changes in auxin responses correlated with proximal-distal specification in the PLB. If auxin transcriptional responses were associated with the specification of the ligule founder cells, high DR5 signal would be expected in the proximal and central regions of the PLB before ligule outgrowth, similar to the localization of PIN1a-YFP. DR5 signal was high in the underlying vasculature, which indicated auxin responses in those regions (Fig. 7). Special care was taken to measure DR5 intensity only in epidermal cells located between vascular bundles, thus excluding the strong signal from underlying vasculature (e.g. areas labeled with a V for vasculature in Fig. 7). In contrast to strong PIN1a-YFP signal (Fig. 7A-E), DR5 signal was weak throughout the entire PLB, and gradually increased after the ligule fringe formed (*F*-test, *P*<0.01; Fig. 7F-P). These data suggest that DR5-related auxin responses do not underlie the specification of the ligule founder cells, although we cannot rule out the possibility that a distinct, DR5-independent subset of auxin responses may occur.

## DISCUSSION

**Fig. 8.**
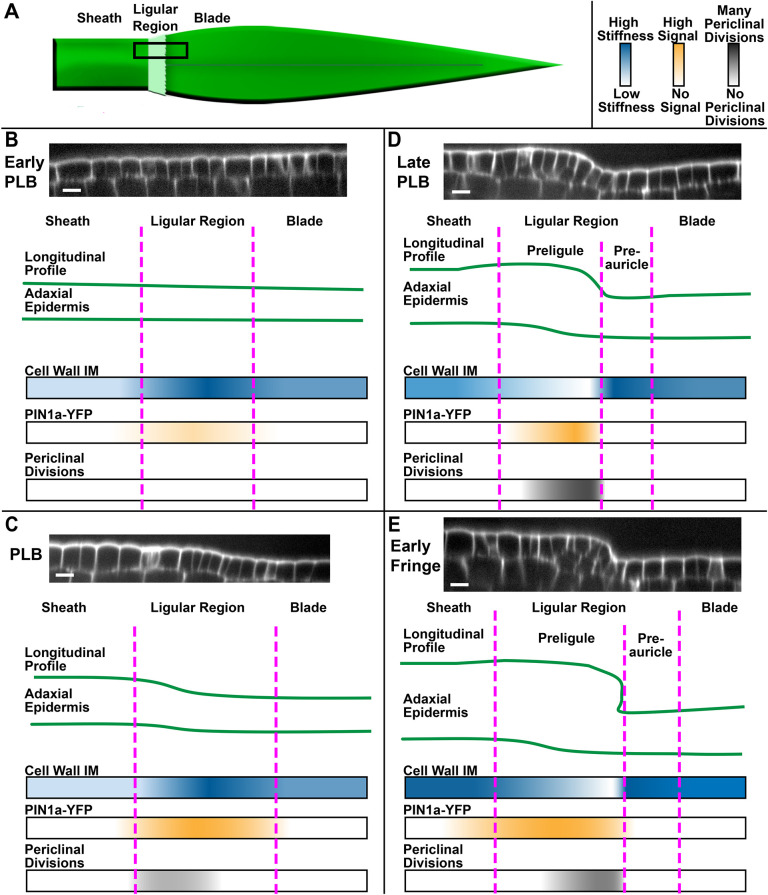
**Summary of patterns observed during early ligule development.** (A) Cartoon of maize leaf primordium and proximodistal zones. Black box indicates the area studied. (B-E) Patterns in epidermal topography are shown using representative confocal micrograph projections and cartoons. Proximodistal patterns in cell wall IM, PIN1a-YFP signal and the position/frequency of periclinal divisions are shown using color gradients. Panels show early PLB stage (B), PLB stage (C), late PLB stage (D) and early fringe stage (E). Scale bars: 15 μm.

A key problem in plant development is understanding how new growth axes are generated distinct from pre-existing growth axes. Establishment of boundaries and boundary-like domains can help facilitate the physical separation of new organs or structures by locally limiting growth, but the mechanisms restricting cell expansion in boundaries are not clear (Bell et al., 2012; Gendron et al., 2012; Lee et al., 2009). In maize, the formation of the ligule is a particularly complex morphogenic process because a thin flap forms entirely from epidermal cells and cleanly diverges from the rest of the epidermis along a well-defined cleft at the ligule-auricle junction. Here, we examine cellular properties across the ligular region during early ligule development (Fig. 8A). In the early PLB (Fig. 8B), PIN1a-YFP localizes throughout the entire PLB, with the strongest signal overlying the vasculature. At this stage, cell division orientation is exclusively anticlinal, epidermal cell depth is uniform and the topography of the leaf surface is nearly flat in the proximodistal direction. Epidermal cell walls are more rigid in the PLB compared with the blade and sheath. In the PLB stage (Fig. 8C), PIN1a-YFP signal becomes stronger and more uniform throughout the entire ligular region, but the protein remains relatively nonpolar at the subcellular level. During the PLB stage, cells in the sheath epidermis and proximal ligular region increase in depth considerably more than the distal ligular region and blade, and periclinal divisions are observed in the proximal ligular region. These changes contribute to the formation of a ridge that forms immediately proximal to the zone with the most rigid cell walls. During the late PLB stage (Fig. 8D), the zone of PIN1a-YFP accumulation narrows, localizing to the nascent ridge. The frequency of periclinal divisions reaches a maximum and the cells on the ridge have softer cell walls. At this stage, the proximal and distal zones of the ligular region differ in epidermal thickness, division plane orientation, cell wall rigidity and PIN1a-YFP accumulation. In the early fringe stage, the soft cells on the more proximal ridge grow over the top of the more distal rigid cells, forming a well-defined cleft (Fig. 8E). The newly separated growth axis of the early ligule fringe then elongates primarily via transverse divisions and cell expansion. Our findings are consistent with the model proposed by Johnston et al. (2014), which was based on expression profiling, that the PLB is partitioned into subdomains before ligule outgrowth, which may predict the distinction between forming ligule and auricle on the adaxial surface. Aside from potential preligule-preauricle specification, there may be additional subdomains that are not currently recognized.

The abrupt shift from anticlinal to periclinal division orientation is a key feature of developmental events in plants. Mechanisms regulating this shift are not well understood. Previous predictive modeling of cell divisions via soap-film minimization showed that the geometry of cells in the late PLB favors periclinal divisions (Martinez et al., 2018). Late PLB cells are small in the epidermal surface, but relatively thick in the depth axis, resulting in a columnar cell shape. Cells tend to divide along the shortest axis, so the Martinez et al. (2018) geometry-based surface minimization model most commonly predicts periclinal divisions in these cells. Our data show that the earliest periclinal divisions in the PLB stage are observed in the proximal and central PLB cells, which have thickened more in the depth axis than the distal cells. Furthermore, the periclinal division rate is highest at the late PLB stage, when cell area at the epidermal surface is the smallest. Differential cell thickening establishes a geometry in the proximal and central PLB cells that favors periclinal division plane orientation.

Our data add to the existing body of nanoindentation and AFM experiments on live plant cells (Bou Daher et al., 2018; Majda et al., 2017; Peaucelle et al., 2011; Routier-Kierzkowska et al., 2012). The dramatic softening of cell walls in the proximal PLB preceding ligule outgrowth is highly reminiscent of AFM experiments in *Arabidopsis*, where biochemical changes and mechanical softening in the cell walls correlate with increased growth (Peaucelle et al., 2011; Bou Daher et al., 2018). The juxtaposition between rigid and soft epidermal cells along a discrete line is conspicuous, and suggests that differential regulation of cell wall properties within adjacent cell populations mechanically contributes to the sharp cleft at the preligule-preauricle junction, reminiscent of earlier studies showing that mechanical patterns contribute to abrupt changes in directional growth at the shoot apex (Selker et al., 1992). PLB cells also exhibit reduced elastic asymmetry between transverse and longitudinal wall segments. Elastic asymmetry was shown to correlate with anisotropic expansion in the *Arabidopsis* hypocotyl (Bou Daher et al., 2018). A shift to isotropic growth is observed during leaf initiation from the SAM peripheral zone (Sassi et al., 2014), so it is possible that a similar trend may occur during early ligule outgrowth. These findings may inform future experiments exploring differences in cell expansion and growth anisotropy during maize leaf development.

We note that the indentation modulus of the cell wall is not a direct indicator of extensibility nor actual cell expansion, which is a plastic irreversible process (Cosgrove, 2016). The cell wall is heterogeneous and materially anisotropic; each cell exists within the structure of multiple tissue layers, and changes in wall chemistry, heterogeneity and degree of plasticity occur during growth and development. Computational modeling could help explore the mechanics of nanoindentation in live plant tissue, and the biological implications of the elastic properties of the cell wall. Finally, more experiments are necessary to determine the cell wall components, remodeling enzymes or other properties underlying the observed differences in rigidity between epidermal regions.

The accumulation of PIN-like genes in the PLB has been previously reported, suggesting a role for auxin transport in ligule development (Johnston et al., 2014; Moon et al., 2013). We observed that the PIN1a-YFP-accumulating zone narrowed significantly during the late PLB stage, becoming restricted to the small PLB cells in the center of the ligular region. This could be consistent with the focusing of auxin toward a convergence point, as it is during leaf initiation (Conklin et al., 2019). However, PIN1a-YFP accumulation in the PLB and ligule was relatively nonpolar, and no obvious DR5 maximum was observed in the PIN1-accumulating domain. This is puzzling because *PIN1a* is an auxin-responsive gene and other auxin-regulated genes, including AUXIN RESPONSE FACTORs (ARFs), SMALL AUXIN UPREGULATED RNAs (SAURs) and GRETCHEN HAGEN3 (GH3) genes, are differentially expressed in the PLB (Johnston et al., 2014). Nonpolar auxin efflux and a lack of DR5 signal are consistent with low auxin transcriptional responses in the PLB, rather than the elevated responses associated with the initiation of many other plant organs. There are 15 PIN genes in maize (Yue et al., 2015), several of which are upregulated in the PLB, such as *PIN5*, *PIN1c* and *SoPIN1* (Johnston et al., 2014). Other PIN proteins could localize differently than our PIN1a-YFP construct. For example, AtPIN1 is involved in polar auxin transport in the epidermis of the *Arabidopsis* meristem during leaf initiation, but in maize this role is filled by SoPIN1, which belongs to the SISTER-OF-PIN1 (SoPIN1) clade, whereas AtPIN1 orthologs ZmPIN1a and ZmPIN1b act in internal tissue layers (Carraro et al., 2006; Li et al., 2019; O'Connor et al., 2014). The expression of *TIR_1*/AFBs, IAAs, and ARFs, and differential affinities for auxin, can affect the sensitivity of auxin signaling in a given region (Vernoux et al., 2011). Although TIR/AFB auxin receptor genes are expressed relatively consistently between the blade, ligular and sheath zones, both ARFs and IAAs are differentially expressed in the PLB (Johnston et al., 2014). It is possible that a distinct subset of auxin responses is activated in the PLB without high *DR5* expression. In particular, *GRMZM2G158359*, a likely ortholog of the transmembrane noncanonical auxin receptor gene *AtTMK1*, is significantly upregulated in the PLB (FDR<0.05, Johnston et al., 2014), suggesting that extracellular auxin could serve a signaling role in the PLB without activating canonical TIR_1/AFB-AuxIAA signaling (Cao et al., 2019; Lin et al., 2021; Xu et al., 2014). With so much complexity governing auxin signaling and responses, the role of auxin in the development of the ligular region remains unclear.

Our data support a model in which the boundary between blade and sheath in the maize leaf is progressively refined in the ligular region, producing two subdomains, as previously proposed based on SEM and gene expression data (Sylvester et al., 1990; Johnston et al., 2014). Shifts in topography, cell growth, division orientation and PIN localization correlate with changes in cell wall biophysical properties in the ligular region. The rigid PLB is partitioned into a soft proximal incipient ligule, and a rigid distal zone, which we propose is the early differentiation of auricle cells on the adaxial surface. These events correlate with ligule outgrowth and presage the development of the auricle between the ligule and blade. How this occurs across the three dimensions of the leaf is intriguing, given that the auricle hinge becomes anatomically unique in all polarity dimensions. Next steps are to refine molecular and cellular changes in the transverse and mediolateral three-dimensions to fully understand how ligule- and auricle-specific cell growth is coordinated.

## MATERIALS AND METHODS

### Plant growth and dissection

Maize plants were grown in two-gallon pots in standard greenhouse conditions (28°C, 16 h light/8 h dark) for 2-4 weeks at the Laramie Research and Extension Center at the Agriculture Experiment Station at the University of Wyoming or in greenhouses under similar conditions at the University of California, Riverside. Maize plants used for imaging included the inbreds B73, Mo17, and plants containing fluorescent markers developed by the Maize Cell Genomics project (http://maize.jcvi.org/cellgenomics/index.php). Maize lines expressing *PIN1a-YFP* and *DR5rev*:*mRFPer* (Gallavotti et al., 2008), *YFP-TUBULIN* and *CFP-TUBULIN* (Mohanty et al., 2009), and *TAN1-YFP* (Wu et al., 2013; Martinez et al., 2017) have been previously described. Transgenic plants were selected by resistance to a solution of 4 g/l glufosinate-ammonium (Basta, Bayer Sciences) in 0.5% Tween applied to the leaf. Plants were genotyped by PCR using primers CYFP LSP1 (5′-agcgcgatcacatggtcct) and PIN4110R (5′-ttcccgaagctgaagtcgtcc) or DR5-870F (5′-tgaagggcgagatcaagatgag) and DR5-1225R (5′-ctcaacacatgagcgaaacc).

For dissections, leaves were sequentially removed from the plant and leaf numbers counted from leaf 1 in toward the SAM. The length of the sheath region was measured with calipers, and the ligule growth stage was assessed by either confocal microscopy or SEM (all stages), or in a separate set of experiments by AFM (below 3.5 mm sheath length). The leaves examined ranged from leaf numbers 4 to 12, depending on plant age and the developmental stage at which the plants were collected. Although the mediolateral position was not strictly controlled, imaging and measurements were collected from the lateral and marginal domains of the leaf primordium, not the central domain (Hay and Hake, 2004).

### Imaging and measuring cell size and division arrays using YFP-TUBULIN lines

Adaxial ligule regions of freshly dissected plants were mounted in water in Rose chambers and micrographs were analyzed for cell area and division plane orientation using ImageJ (http://rsbweb.nih.gov/ij/). All imaged cells in the ligular region that had visible YFP-labeled preprophase bands, mitotic spindles or phragmoplasts were considered. Angles of preprophase bands, spindles or phragmoplasts were classified as anticlinal transverse, anticlinal longitudinal, periclinal or oblique relative to the long axis of the leaf, as shown in Fig. 2A. We imaged 3-5 leaves expressing YFP-Tubulin per stage. To calculate cell area in each leaf, three boxes each encompassing 20-100 cells were drawn spanning the PLB or over a portion of the elongating ligule in the confocal micrographs, and the number of cells in each box was counted. Areas of the boxes were divided by the number of cells in each box to calculate the average cell area.

At the PLB and late PLB stages, the relative position of actively dividing cells within the PLB was determined using ImageJ. First, the proximal and distal extremities of the ligular region were traced according to differences in cell size and shape (Fig. 4A). Then, actively dividing cells, as indicated by the presence of a preprophase band labeled with either YFP-TUBULIN or TAN-YFP, were located within the PLB (Movie 1). Their relative position was calculated by measuring the distance from the proximal end of the PLB to the center of the dividing cell, and then from the proximal end to the distal end of the PLB, and dividing the former value by the latter. This generates values ranging from 0 at the proximal end of the PLB to 1 at the distal end (Fig. 4A).

### Confocal microscopy

Images were acquired on two spinning disk confocal microscopes. The EM-CCD camera (ImagEM, Hamamatsu) was mounted on an IX71 stand equipped with a spinning-disc confocal head (CSU-X1; Yokogawa). An LMM5 laser launch was used to provide illumination (Spectral Applied Research). Laser lines of 488 and 561 nm were used to excite PIN1a-YFP, YFP-TUBULIN, TAN1-YFP and *DR5rev*:*mRFPer* with band pass filters ET525/50M and ET595/50M (Chroma Technology), respectively. Some image acquisition was performed using Metamorph 7.7 software (Molecular Devices). Images were acquired using 20× (0.85 NA) and 40× (1.30 NA) oil Olympus objectives. For additional samples in the early PLB, PLB and late PLB stages of ligule development, the dissected ligular region was stained with 10 μg/ml propidium iodide (PI) for 10 min and mounted in water. Confocal scans were collected using the 40× objective lens through the epidermis with a *z*-step of 0.2 μm using a Hamamatsu 9100C EM-CCD camera mounted on a Nikon Ti stand with a spinning disc confocal head (CSU-W1, Yokogawa) and a 40× water (1.1 NA) Nikon water objective. PI was excited at 561 nm and collected at 620/20 nm. TAN-YFP and YFP-TUBULIN were excited at 514 nm and collected at 540/30 nm, and CFP-TUBULIN was excited at 445 nm and collected at 480/40 nm.

### Scanning electron microscopy

We used 2-, 3- and 4-week-old B73 leaf samples for SEM to characterize ligule stages. Sheath lengths were measured and the ligular region was excised with a scalpel, mounted with two-sided tape and loaded directly into the sample chamber of the tabletop electron microscope (Hitachi TM-1000), with included software used to acquire images.

### Image and statistical analysis

Image analysis was performed using ImageJ, FIJI (ImageJ) or Metamorph v. 7.7. Data were analyzed in Excel and Access (Microsoft Office) and graphs produced in GraphPad (Prism) and R. For measuring PIN1a-YFP polarity (Fig. 5M), 30 cells at each stage from four different plants were used for analysis. PIN1a-YFP fluorescence intensity measurements were performed by scanning through a *z*-stack of an entire epidermal cell. The plane with the highest fluorescence intensity value was selected at each side of the randomly selected cell. A 1-pixel-thick line was drawn across each side of the cell cortex and average intensity values were recorded. Ratios were calculated by dividing the highest average intensity value by the lowest for each analyzed cell in Excel and the error bars are 95% confidence intervals.

For measuring DR5 fluorescence intensity, images were background subtracted using a rolling ball radius of 50 pixels and normalized. A box was drawn between veins to avoid fluorescence of underlying vasculature (see Fig. 7F). Average fluorescence intensity was recorded from the boxed area of at least 1000 square pixels from a 5 µm deep maximum projection. For DR5 expression, three plants were used per stage and standard error bars are shown in Fig. 7. The average intensity values from areas between vascular bundles of three samples in a single plant were normalized by dividing each value by the lowest average DR5 intensity value. Significance tests comparing the distribution of PIN1a-YFP fluorescence intensity ratios were performed using the non-parametric Kolmogorov–Smirnov (KS) test.

Cell depth was measured for PI-stained leaves dissected from three plants in the early PLB, PLB and late PLB stages using ImageJ. The image stack was projected as an orthoslice and the thicknesses of cells were measured at transverse wall segments by counting the number of *z*-steps between the top and bottom of the wall segment. Statistical differences were assessed via the non-parametric Kruskal–Wallis test, with a Dunn's post-hoc for pairwise comparisons.

For the distribution of anticlinal and periclinal divisions in the ligular region, the sheathward and bladeward extremities of the ligular region were determined by differences in cell size and shape in confocal scans of TAN-YFP plants stained with PI. For the purpose of this analysis only, the sheathward limit of the ligular region was relative position 0, while the bladeward limit was relative position 1. The relative proximodistal position of cells undergoing anticlinal and periclinal divisions was determined. One sample chi-square tests for variance were used to determine whether a certain division type was uniformly distributed or confined to a particular subdomain of the ligular region.

Representative confocal *z*-stacks at each stage were analyzed using MorphoGraphX to extract cell area, surface curvature, and cell depth data as described by the user manual and previous experiments (Kierzkowski et al., 2012; http://www.MorphoGraphX.org). Stacks were processed using a Gaussian blur with a sigma of 0.3 µm. The epidermal surface was found using Edge Detect, with the proper threshold determined for each scan individually, and the surfaces were smoothed using Fill Holes, Erode, Dilate and Smooth functions as necessary. Meshes of the surfaces were generated using the Marching Cubes function at a cube size of 1 µm, and the *z*-stacks were projected onto the resulting meshes. Cells were seeded manually, then Watershed Segmentation was performed at the default threshold. Cell geometric data was calculated and heatmaps of cell size were projected onto the segmented mesh. Average curvatures of the surfaces were calculated at a neighborhood value of 50 µm and projected onto the segmented surfaces as heatmaps. The edges of the curvature maps were deleted owing to errors in the curvature calculations near the edges. Curvature and cell area were plotted together on the same surface by plotting cell area as heatmaps, then plotting tissue curvatures at the center of each cell as linear vectors indicating the direction, sign and magnitude of maximum curvature. For cell depth, blurred *z*-stacks were used for auto-seeded ITK watershed segmentation, at threshold values that were optimized for each sample. The segmentation was corrected manually by comparing with the original *z*-stack. Incomplete cells around the edges were deleted, as were many of the underlying cells. Cell meshes of the remaining cells were created using the 3D Marching Cubes function at a cube size of 1 µm. The cell meshes were analyzed in 3D and cell depths were projected as a heatmap.

When analyzing AFM data, the difference in sheath length between the two observed tissue-level mechanical patterns was assessed via a Mann–Whitney *U*-test. For manual resampling, at least 50 indentations were used per wall category per tissue zone per sample to calculate average IM values. After manual resampling, global variation in IM with respect to wall category and tissue zone were assessed via Kruskal-Wallis tests. Then, pairwise Wilcoxon signed rank tests were performed at significance levels of *P*<0.05 and *P*<0.01, using the *W*-statistic. Variation in IM with respect to developmental stage was assessed via Mann–Whitney *U*-tests.

### Atomic force microscopy

Developing ligules were dissected as described above and sheath length was measured using electronic calipers. To eliminate turgor pressure and only consider cell wall mechanical properties, leaves were plasmolyzed before being measured. The samples were quickly placed in 0.55 M mannitol solution for at least 15 min to induce plasmolysis, before being affixed to a microscope slide using double-sided tape. Additional mannitol solution was used to immerse the sample and pre-wet the probe.

AFM was performed using a JPK NanoWizard 4a AFM in force mapping mode, at the California NanoSystems Institute at UCLA. Indentations were performed with a constant maximum force of 500 nN, with extend and retract times of 0.1 s, at a spatial resolution of at least one indentation per 2 μm. The probes used were PPP-NCL probes with a 10 nm pyramidal tip, with an average force modulus of 45 N/m. Each tip was calibrated separately to accommodate slight differences.

Data were processed using the JPKSPM software. The raw indentation data were converted into IM using the Hertzian contact model as previously described (Peaucelle et al., 2011). Because the maximum scan area was too small to adequately sample all epidermal regions in a single scan, multiple overlapping scans were measured, processed and manually reassembled by identifying cell walls in the overlapping areas. To quantify IM along the longitudinal axis of the leaf, regional IM values were averaged using a sliding window approach. For a given leaf, a 25 μm-wide rectangle was drawn and repositioned along the longitudinal axis until the local maximum for average IM was located in the ligular region. This position was designated relative position 0 and the average IM for that bin was set as 1. Regional averages for IM were then measured along the proximodistal axis in 25 μm-wide bins, with a 12.5 μm step between bins. Position and average IM were normalized to the local maximum for each leaf measured. Manual resampling of the scans was performed using a custom script in MATLAB (github.com/mathworks). Force maps were projected as a heatmap and at least 50 pixels within each epidermal zone and cell wall category were selected and averaged for each sample. We thoroughly sampled each cell in each tissue region, resampling at least five indentations per wall category per cell.

## Supplementary Material

10.1242/develop.201608_sup1 Supplementary information
