# Supplementary material for: The maize preligule band is subdivided into distinct domains with contrasting cellular properties prior to ligule outgrowth
Source: Development. 2023 Oct 30;150(21):dev201608. doi: 10.1242/dev.201608 (PMC10629682; doi:10.1242/dev.201608)
Supplement: Supplementary information [file develop-150-201608-s1.pdf]

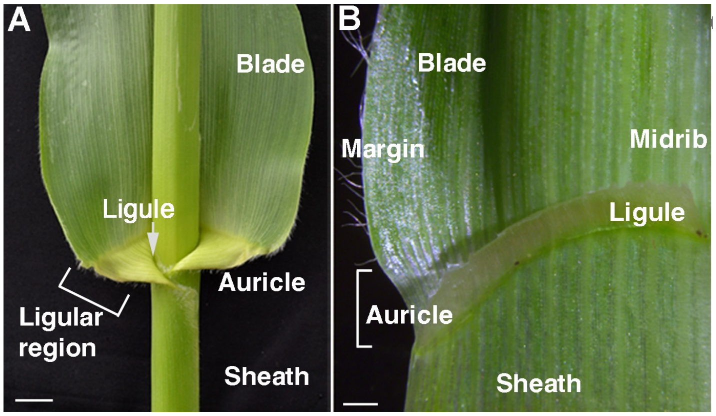

Fig. S1. Maize leaf structure.

The maize leaf is composed of a distal blade and proximal sheath separated at the ligular region (bracket), consisting of a ligule and auricle. Scale bar = 2 cm. (B) The leaf is cut at the midrib to expose the adaxial view of the ligular region. Scale bar = 1 mm.

Development • Supplementary information

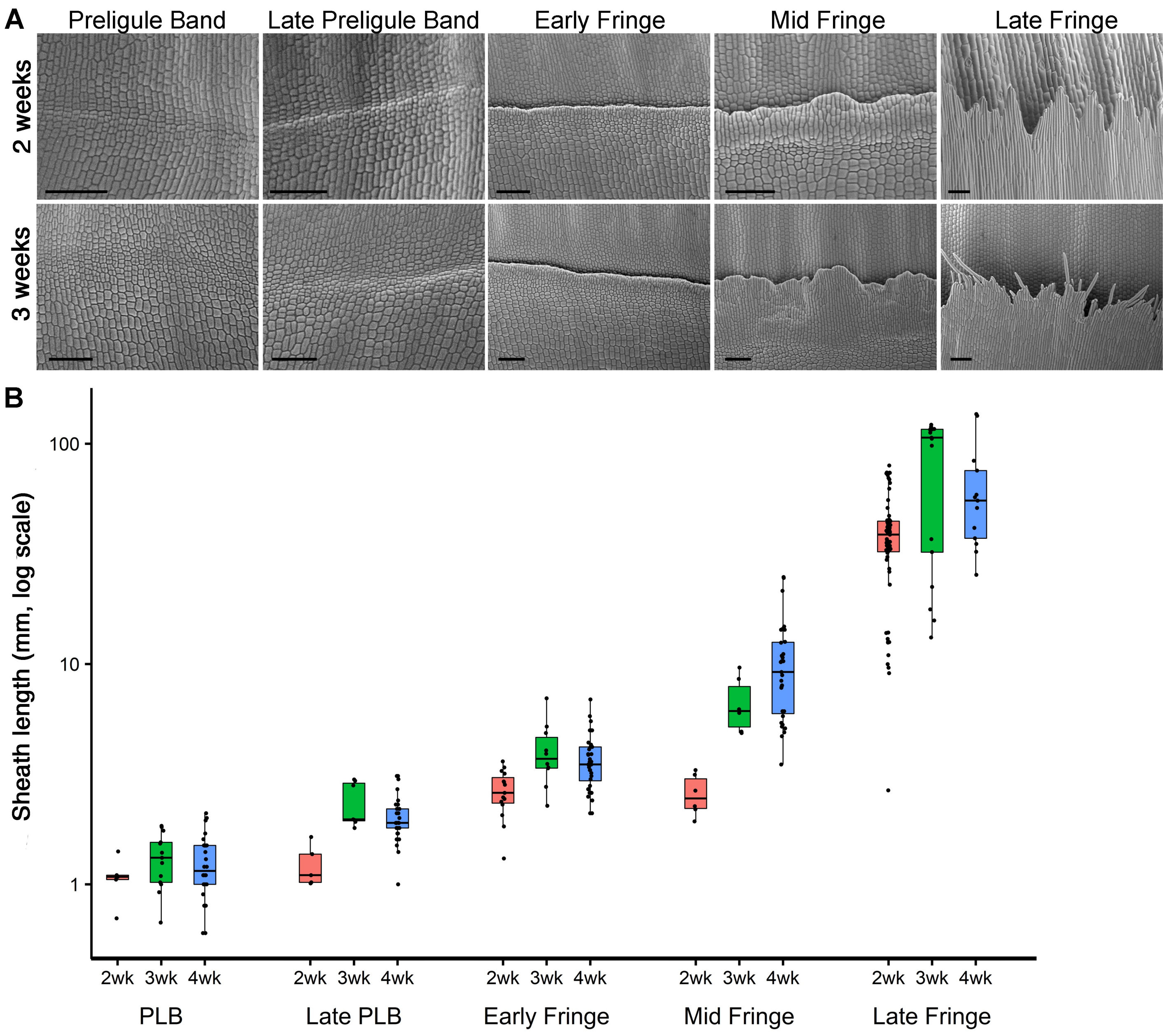

Fig. S2. Stages of ligule development relative to sheath height in 2-, 3-, and 4-week-old plants.

Scanning electron micrographs of sequentially dissected leaves of 2- and 3-week-old maize plants. (B) Stages of ligule development in 2-, 3-, and 4-week-old maize plants relative to sheath length. Scale bar = 100 µm.

Development • Supplementary information

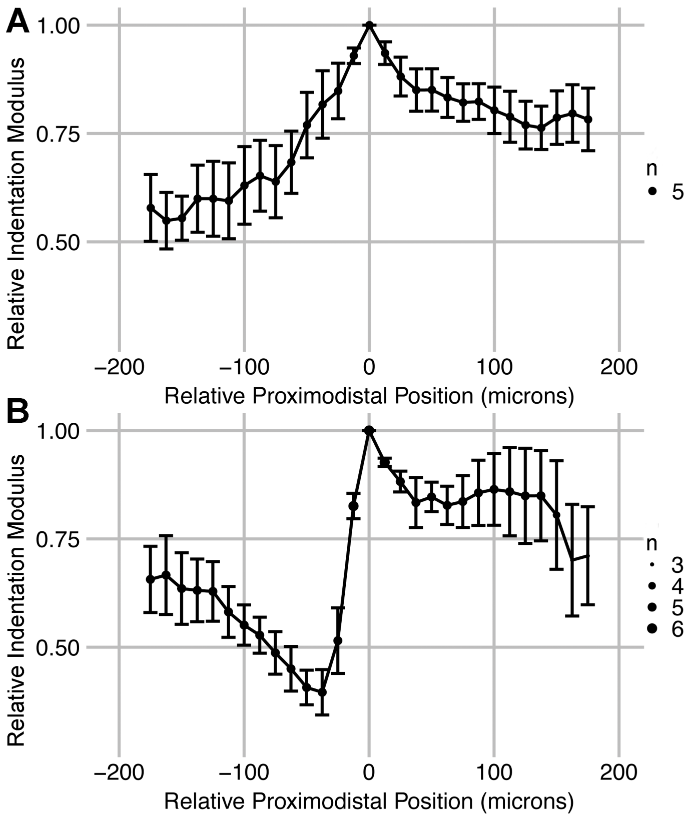

Fig. S3. AFM force mapping sliding window analysis of Mo17 leaves.

Sliding window regional averages in IM. Data normalized to the local maximum in the PLB, set as position=0, relative IM=1. Top panel is the average of all Mo17 plants (n=5) exhibiting the early mechanical pattern. Bottom panel is the average of all Mo17 plants (n=6) exhibiting the late mechanical pattern. Error bars indicate standard error.

Development • Supplementary information

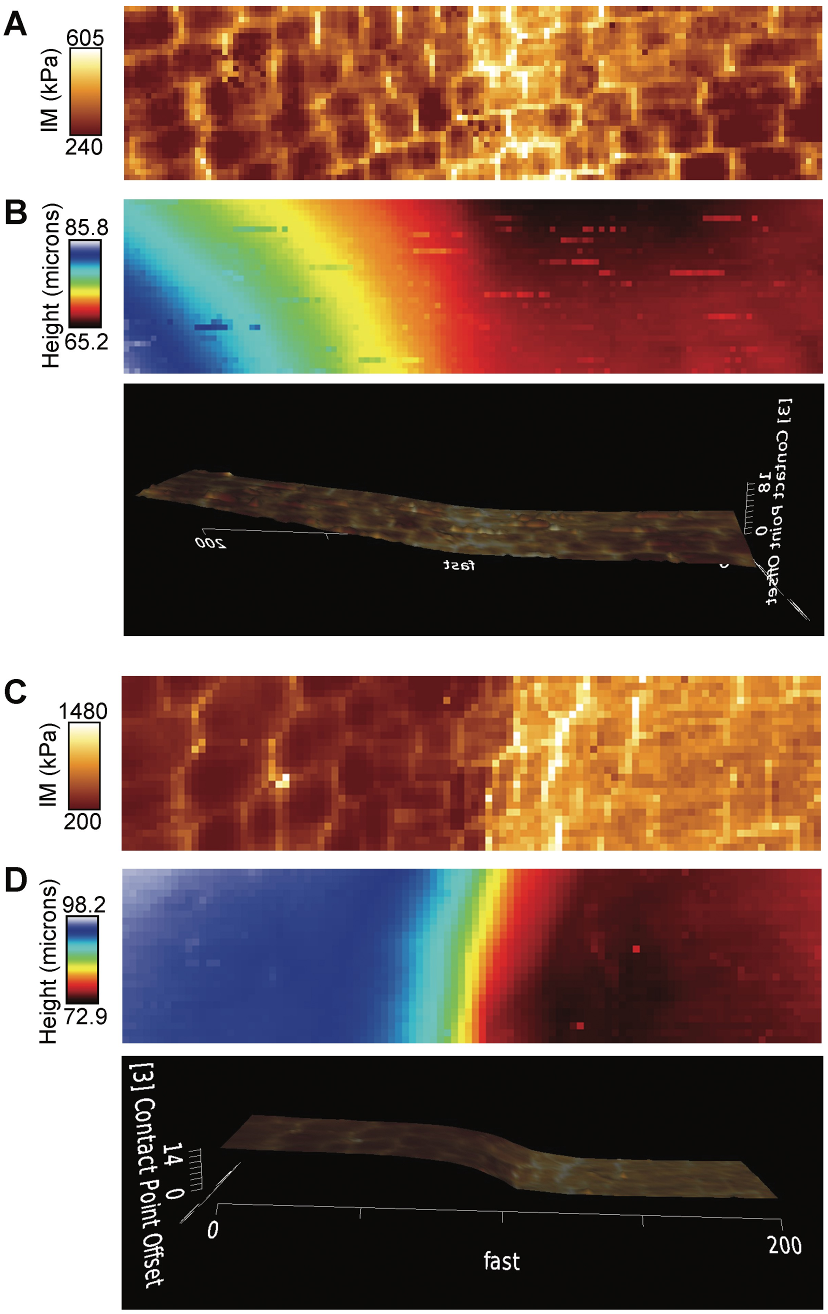

Fig. S4. Mechanical transition within PLB relative to topography.

(A) AFM scan of a leaf in the PLB stage, sheath length 0.6mm. Resolution is 1.6 μm per pixel. (B) Topography of the same sample as (A). This height map was first normalized along the mediolateral axis to highlight topography in the proximodistal direction. Height portrayed via both heatmap and 3D projection (C) AFM scan of a leaf in the late PLB stage, sheath length 2.0mm. Resolution is 2.0 μm per pixel. (D) Topography of the same sample as (C). Scale bars = 50 μm.

Development • Supplementary information

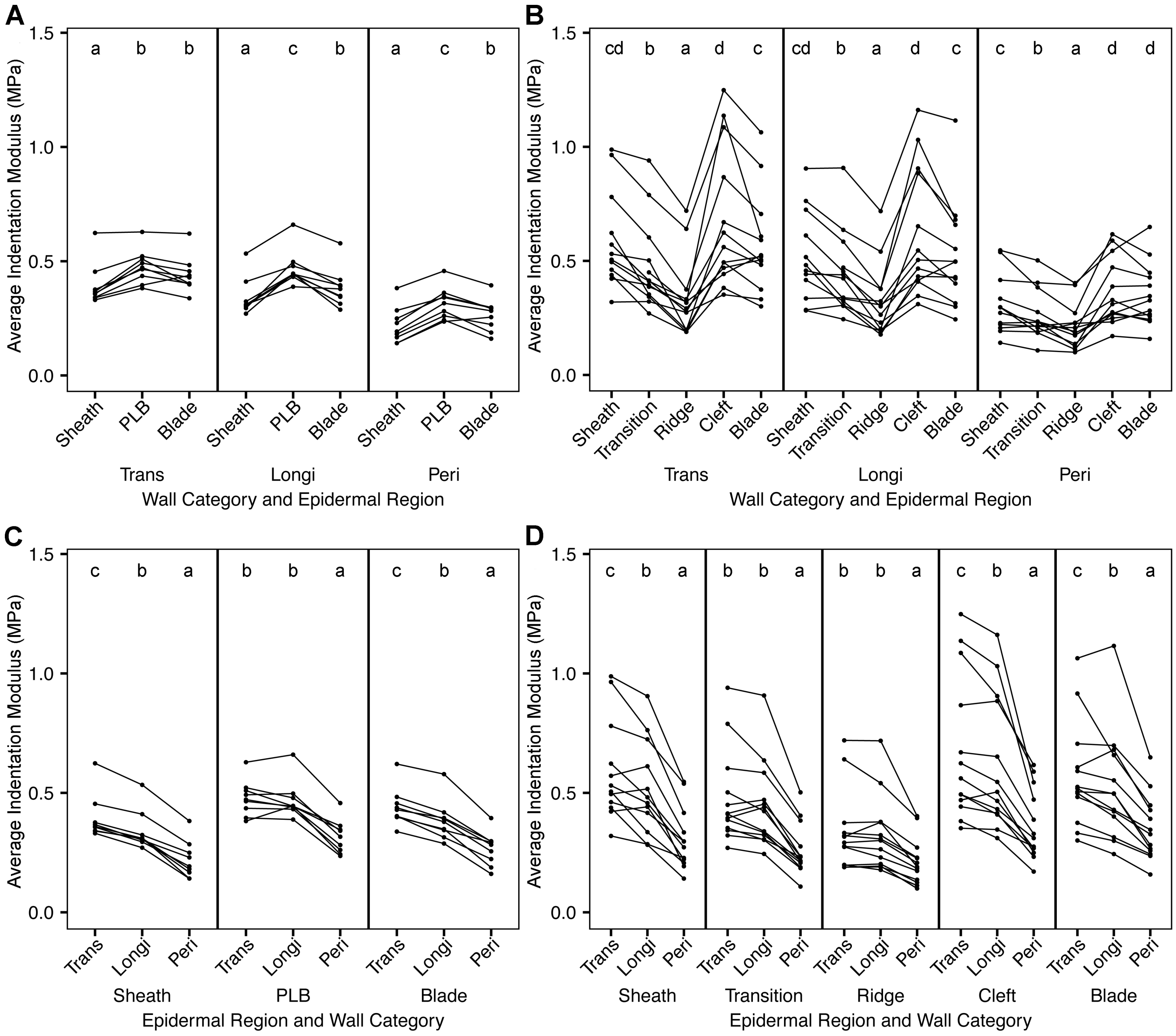

Fig. S5. Manual resampling reveals regional and subcellular patterns in IM.

IM was resampled for each wall category in each epidermal region of each sample. Samplings from different wall categories or tissue zones in the same leaf are connected with lines. Each dot indicates the average IM of at least 50 indentations from a particular wall category in a particular epidermal zone in one leaf. (A) All early-stage B73 samples, grouped by epidermal zone. (B) All late-stage B73 samples, grouped by epidermal zone. (C,D) Same data but instead grouped by wall category. Statistical analysis was performed independently for each panel and subpanel. Statistical significance was determined via Kruskall-Wallis test followed by pairwise Wilcoxon signed rank tests using the *W*-value at an alpha of $p<0.05$.

Table S1. Cell division orientation during ligule developmental stages

Statistical differences for cell division % were determined by MANOVA testing for an effect of developmental stage and division orientation on % divisions, followed by one-way ANOVAs testing for differences with respect to stage and orientation separately. Latin letters indicate significant differences in the % of different division orientations in each stage; Greek letters indicate significant differences for one division type between stages.

Developmental stage	n Individual leaves measured per stage	Sheath length range in mm	Cell size (μm^2 +/- S.E.)	Total mitotic cells observed	Anticlinal longitudinal division (% +/- S.E.)	Anticlinal transverse divisions (% =/- S.E.)	Periclinal divisions (% +/- S.E.)	Oblique divisions (% +/- S.E.)
Early Preligule band	4	0.8 - 1.3	158 +/- 9<br>n=414	77	**52 +/- 9%**<br>b<br>γ	49 +/- 8%<br>b<br>αβ	0%<br>a<br>α	3 +/- 2%<br>a<br>α
Preligule band	3	1.5 - 2.1	134 +/- 9<br>n=315	59	38 +/- 3%<br>b<br>βγ	**45 +/- 5%**<br>b<br>αβ	10 +/- 4%<br>a<br>αβ	5 +/- 3%<br>a<br>α
Late Preligule band	5	1.8 - 3.1	105 +/- 6<br>n=759	132	18 +/- 2%<br>b<br>αβ	31 +/- 2%<br>c<br>α	**46 +/- 1%**<br>d<br>δ	4 +/- 2%<br>a<br>α
Early fringe	3	2.5 - 4.4	186 +/- 16<br>n=304	62	22 +/- 1%<br>b<br>αβ	**48 +/- 1%**<br>c<br>αβ	27 +/- 2%<br>b<br>γ	2 +/- 2%<br>a<br>α
Mid fringe	3	5.4 - 12.5	450 +/- 145<br>n=539	45	9 +/- 5%<br>a<br>α	**71 +/- 9%**<br>b<br>β	16 +/- 5%<br>a<br>βγ	2 +/- 2%<br>a<br>α
Late fringe	3	35.1 - 57.5	1511 +/- 147<br>n=246	0	n/a	n/a	n/a	n/a

Development • Supplementary information

Table S2. Pairwise comparisons of average IM between epidermal zones via Wilcoxon signed rank test using the *W*-statistic

Comparison (Epidermal Tissue Zones)	Wall Category	Average IM Zone 1 +/- s.d. (MPa)	Average IM Zone 2 +/- s.d. (MPa)	*W*-value	Significance level
Early Sheath v. Early PLB	Trans	0.394 +/- 0.093	0.478 +/- 0.074	0	p<0.01
	Longi	0.340 +/- 0.082	0.470 +/- 0.077	0	p<0.01
	Peri	0.219 +/- 0.078	0.316 +/- 0.070	0	p<0.01
Early Sheath v. Early Blade	Trans	0.394 +/- 0.093	0.441 +/- 0.079	1	p<0.01
	Longi	0.340 +/- 0.082	0.384 +/- 0.084	0	p<0.01
	Peri	0.219 +/- 0.078	0.265 +/- 0.069	0	p<0.01
Early PLB v. Early Blade	Trans	0.478 +/- 0.074	0.441 +/- 0.079	6	NS
	Longi	0.470 +/- 0.077	0.384 +/- 0.084	0	p<0.01
	Peri	0.316 +/- 0.070	0.265 +/- 0.069	1	p<0.01
Late Sheath v. Late Transition	Trans	0.591 +/- 0.213	0.475 +/- 0.194	1	p<0.01
	Longi	0.518 +/- 0.196	0.443 +/- 0.180	9	p<0.05
	Peri	0.308 +/- 0.131	0.260 +/- 0.107	5	p<0.01
Late Sheath v. Late Ridge	Trans	0.591 +/- 0.213	0.332 +/- 0.167	0	p<0.01
	Longi	0.518 +/- 0.196	0.323 +/- 0.156	0	p<0.01
	Peri	0.308 +/- 0.131	0.211 +/- 0.097	7	p<0.01
Late Sheath v. Late Cleft	Trans	0.591 +/- 0.213	0.679 +/- 0.304	22	NS
	Longi	0.518 +/- 0.196	0.620 +/- 0.281	18	NS
	Peri	0.308 +/- 0.131	0.362 +/- 0.146	13	p<0.05
Late Sheath v. Late Blade	Trans	0.591 +/- 0.213	0.571 +/- 0.218	25	NS
	Longi	0.518 +/- 0.196	0.524 +/- 0.230	34	NS
	Peri	0.308 +/- 0.131	0.351 +/- 0.134	13	p<0.05
Late Transition v. Late Ridge	Trans	0.475 +/- 0.194	0.332 +/- 0.167	0	p<0.01
	Longi	0.443 +/- 0.180	0.323 +/- 0.156	0	p<0.01
	Peri	0.260 +/- 0.107	0.211 +/- 0.097	9	p<0.01

Development • Supplementary information

Late Transition v. Late Cleft	Trans	0.475 +/- 0.194	0.679 +/- 0.304	0	p<0.01
	Longi	0.443 +/- 0.180	0.620 +/- 0.281	0	p<0.01
	Peri	0.260 +/- 0.107	0.362 +/- 0.146	1	p<0.01
Late Transition v. Late Blade	Trans	0.475 +/- 0.194	0.571 +/- 0.218	0	p<0.01
	Longi	0.443 +/- 0.180	0.524 +/- 0.230	0	p<0.01
	Peri	0.260 +/- 0.107	0.351 +/- 0.134	0	p<0.01
Late Ridge v. Late Cleft	Trans	0.332 +/- 0.167	0.679 +/- 0.304	0	p<0.01
	Longi	0.323 +/- 0.156	0.620 +/- 0.281	11	p<0.05
	Peri	0.211 +/- 0.097	0.362 +/- 0.146	0	p<0.01
Late Ridge v. Late Blade	Trans	0.332 +/- 0.167	0.571 +/- 0.218	0	p<0.01
	Longi	0.323 +/- 0.156	0.524 +/- 0.230	0	p<0.01
	Peri	0.211 +/- 0.097	0.351 +/- 0.134	0	p<0.01
Late Cleft v. Late Blade	Trans	0.679 +/- 0.304	0.571 +/- 0.218	12	p<0.05
	Longi	0.620 +/- 0.281	0.524 +/- 0.230	7	p<0.01
	Peri	0.362 +/- 0.146	0.351 +/- 0.134	38	NS

Development • Supplementary information

Table S3. Pairwise comparisons of average IM between wall categories via Wilcoxon signed rank test using the *W*-statistic

Comparison (Wall Categories)	Tissue Zone	Category 1 Average IM +/- s.d. (MPa)	Category 2 Average IM +/- s.d. (MPa)	*W*-value	Significance level
Early Timepoint, Trans v. Longi	Sheath	0.394 +/- 0.093	0.340 +/- 0.082	0	p<0.01
	PLB	0.478 +/- 0.074	0.470 +/- 0.077	15	NS
	Blade	0.441 +/- 0.079	0.384 +/- 0.084	0	p<0.01
Early Timepoint, Trans v. Peri	Sheath	0.394 +/- 0.093	0.219 +/- 0.078	0	p<0.01
	PLB	0.478 +/- 0.074	0.316 +/- 0.070	0	p<0.01
	Blade	0.441 +/- 0.079	0.265 +/- 0.069	0	p<0.01
Early Timepoint, Longi v. Peri	Sheath	0.340 +/- 0.082	0.219 +/- 0.078	0	p<0.01
	PLB	0.470 +/- 0.077	0.316 +/- 0.070	0	p<0.01
	Blade	0.384 +/- 0.084	0.265 +/- 0.069	0	p<0.01
Late Timepoint, Trans v. Longi	Sheath	0.591 +/- 0.213	0.518 +/- 0.196	0	p<0.01
	Transition	0.475 +/- 0.194	0.443 +/- 0.180	19	NS
	Ridge	0.332 +/- 0.167	0.323 +/- 0.156	31	NS
	Cleft	0.679 +/- 0.304	0.620 +/- 0.281	7	p<0.01
	Blade	0.571 +/- 0.218	0.524 +/- 0.230	16	p<0.05
Late Timepoint, Trans v. Peri	Sheath	0.591 +/- 0.213	0.308 +/- 0.131	0	p<0.01
	Transition	0.475 +/- 0.194	0.260 +/- 0.107	0	p<0.01
	Ridge	0.332 +/- 0.167	0.211 +/- 0.097	0	p<0.01
	Cleft	0.679 +/- 0.304	0.362 +/- 0.146	0	p<0.01
	Blade	0.571 +/- 0.218	0.351 +/- 0.134	0	p<0.01
Late Timepoint, Longi v. Peri	Sheath	0.518 +/- 0.196	0.308 +/- 0.131	0	p<0.01
	Transition	0.443 +/- 0.180	0.260 +/- 0.107	0	p<0.01
	Ridge	0.323 +/- 0.156	0.211 +/- 0.097	0	p<0.01
	Cleft	0.620 +/- 0.281	0.362 +/- 0.146	0	p<0.01
	Blade	0.524 +/- 0.230	0.351 +/- 0.134	0	p<0.01

Table S4. Pairwise comparisons of average IM between early and late mechanical stages via Mann-Whitney U-Test

Comparison (Early v Late)	Wall Category	Average IM Early +/- S.D. (MPa)	Average IM Late +/- S.D. (MPa)	*z*-score	p-value
Early Sheath v. Late Sheath	Transverse	0.394 +/- 0.093	0.591 +/- 0.213	2.452	**0.014 ***
	Longitudinal	0.340 +/- 0.082	0.518 +/- 0.196	2.168	**0.030 ***
	Periclinal	0.219 +/- 0.078	0.308 +/- 0.131	1.670	0.095
Early PLB v. Late Transition	Transverse	0.478 +/- 0.074	0.475 +/- 0.194	-1.135	0.254
	Longitudinal	0.470 +/- 0.077	0.443 +/- 0.180	-1.202	0.230
	Periclinal	0.316 +/- 0.070	0.260 +/- 0.107	-2.003	**0.045 ***
Early PLB v. Late Ridge	Transverse	0.478 +/- 0.074	0.332 +/- 0.167	-2.671	**0.008 ****
	Longitudinal	0.470 +/- 0.077	0.323 +/- 0.156	-2.738	**0.006 ****
	Periclinal	0.316 +/- 0.070	0.211 +/- 0.097	-2.604	**0.009 ****
Early PLB v. Late Cleft	Transverse	0.478 +/- 0.074	0.679 +/- 0.304	1.402	0.161
	Longitudinal	0.470 +/- 0.077	0.620 +/- 0.281	0.734	0.465
	Periclinal	0.316 +/- 0.070	0.362 +/- 0.146	0.334	0.741
Early Blade v. Late Blade	Transverse	0.441 +/- 0.079	0.571 +/- 0.218	1.602	0.110
	Longitudinal	0.384 +/- 0.084	0.524 +/- 0.230	1.803	0.072
	Periclinal	0.265 +/- 0.069	0.351 +/- 0.134	1.269	0.204

Development • Supplementary information

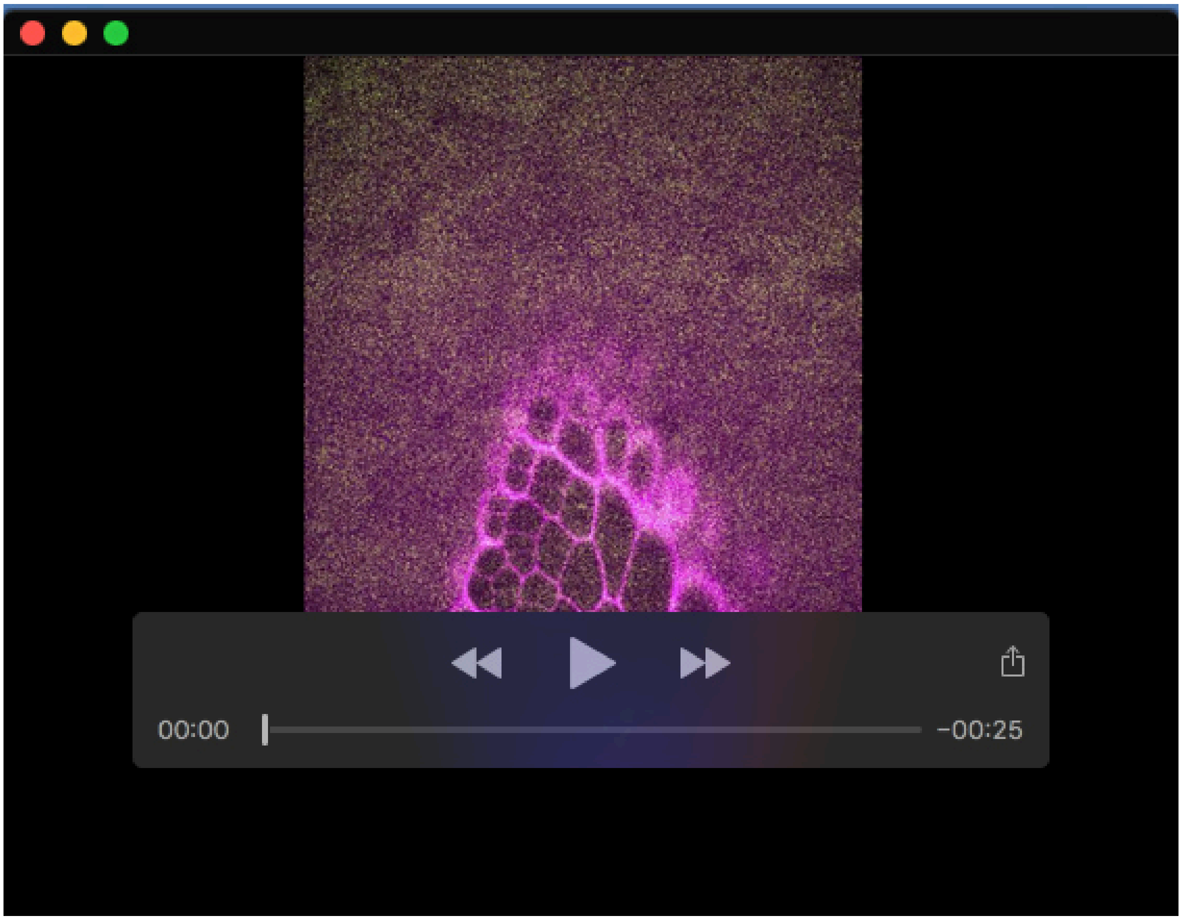

Movie 1. Visualizing periclinal cell divisions.

Leaves from plants expressing TAN-YFP were dissected, stained with propidium iodide (PI), and imaged via confocal microscopy. This movie is made from a sequence of Z-slices from a representative scan. PI is visualized with the magenta channel and TAN-YFP is visualized with the green channel. Periclinally-oriented preprophase bands are visible as green rings running around the circumference of the cell as the focal plane passes through. Two periclinally dividing cells are indicated with arrows.
