# Supplementary material for: The maize preligule band is subdivided into distinct domains with contrasting cellular properties prior to ligule outgrowth
Source: Development. 2023 Oct 30;150(21):dev201608. doi: 10.1242/dev.201608 (PMC10629682; doi:10.1242/dev.201608)
Supplement: Supplementary file 1 [file develop-150-201608_review_history.pdf]

The maize preligule band is subdivided into distinct domains with contrasting cellular properties prior to ligule outgrowth

Wesley R. Neher, Carolyn G. Rasmussen, Siobhan A. Braybrook, Vladimir Lažetić, Claire E. Stowers, Paul T. Mooney, Anne W. Sylvester and Patricia S. Springer
DOI: 10.1242/dev.201608

Editor: Dominique Bergmann

Review timeline

Original submission:	5 February 2023
Editorial decision:	9 March 2023
First revision received:	25 June 2023
Accepted:	28 July 2023

Original submission

First decision letter

MS ID#: DEVELOP/2023/201608

MS TITLE: The maize preligule band is subdivided into distinct domains with contrasting cellular properties prior to ligule outgrowth

AUTHORS: Wesley Neher, Carolyn G. Rasmussen, Siobhan A Braybrook, Vladimir Lazetic, Claire E. Stowers, Paul T. Mooney, Anne W. Sylvester, and Patricia S. Springer

I have now received all the referees reports on the above manuscript, and have reached a decision. The referees' comments are appended below, or you can access them online: please go to BenchPress and click on the 'Manuscripts with Decisions' queue in the Author Area.

The overall evaluation is positive and we would like to publish a revised manuscript in Development, provided that the referees' comments can be satisfactorily addressed. You will see that no new experiments are required, but there are places where additional data analysis will deepen the story. In addition, that each of the reviewers has made suggestions about data presentation and that could make your work more easily accessible to readers. I encourage you to incorporate these suggestions, particularly those from Rev 3 about narrative flow and figure organization.

Please attend to all of the reviewers' comments in your revised manuscript and detail them in your point-by-point response. If you do not agree with any of their criticisms or suggestions explain clearly why this is so. If it would be helpful, you are welcome to contact us to discuss your revision in greater detail. Please send us a point-by-point response indicating your plans for addressing the referee's comments, and we will look over this and provide further guidance.

Reviewer 1

Advance summary and potential significance to field

In this paper Neher and collaborators explore maize ligule development. Using SEM and CLSM they characterize different stages of ligule morphogenesis including changes in cell topology, cell sizes,

© 2023. Published by The Company of Biologists under the terms of the Creative Commons Attribution License (https://creativecommons.org/licenses/by/4.0/).

and cell division distribution. They also observe patterns of selected auxin exporters and auxin responses. Finally, they correlate changes in cell characteristics with the mechanical properties of cell walls measured with AFM indentations. This paper is generally easy to follow, confirms many events previously observed during ligule development (e.g. geometrical changes), and brings some new insight into this process (e.g., characterization of mechanical properties). The conclusions are well supported even if some of the results could be presented in more clear and informative ways (see comments below). The AFM data are convincing and represent the most significant advancement of this paper.

Comments for the author

Below are some comments that, in my opinion, should be considered before the publication of this paper:

1. Figure 1 could be improved by providing:
   a. Heat maps of cell sizes at different developmental stages; This could be also useful to better visualize the decrease in cell sizes observed during early boundary formation (Table S1). Authors have SEM and CSLM data, which should allow it to be easily generated.
   b. Heat maps of surface curvature. This could help better distinguish early-stage PLB from late PLB.

2. Why the early PLB stage is not included in Figure 1?

3. Lines 221-223: “These results show that spatially constrained divisions initially produce the ligule fringe, with cell expansion driving growth in the later stages.” – how do authors know that at early stages cellular growth is less important? It is possible that during the initiation of PLB, the growth is fast but is accompanied by a lot of divisions leading to a decrease in cell sizes.

4. Figure 3. It would be helpful to include representative confocal images (optical sections) of PLB at different developmental stages to visualize the thickening of the epidermis (similar to what is shown in the last figure). Representative images of the cell divisions should also be included.

5. Lines 241-243: “Confocal projections suggested that differential thickening likely also occurs in underlying cell layers; however, reduced signal prevented measuring cell thickness in deeper layers.” If mentioned, then please show the images.

6. Figure 3: Statistically significant differences should be indicated.

7. Lines 269-278: Most of this text should be moved to the method section. It breaks the flow of the story.

8. I am a bit surprised that the PLB regions seem to be the stiffest at the early stages. This is the region where cell divisions are the most intensive. It would be great to correlate the mechanical properties measured with AFM with the cellular growth rates at different developmental stages during ligule initiation.

9. Lines 445-449: “The dramatic softening of cell walls in the proximal PLB preceding ligule outgrowth is highly reminiscent of data from the Arabidopsis SAM, where biochemical changes and mechanical softening in the cell walls of the subepidermal cell layers precede the outgrowth of leaf primordia (Peaucelle et al., 2011).” – I am not sure if the softening of the epidermal layer can be compared with subepidermal softening and between such distinct systems. Also the softening of internal layers in the SAM was reported in incipient primordia
– authors do not observe softening preceding PLB formation.

© 2023. Published by The Company of Biologists under the terms of the Creative Commons Attribution License (https://creativecommons.org/licenses/by/4.0/).

Reviewer 2

Advance summary and potential significance to field

The authors present a beautiful description of the anatomical, biomechanical, and hormonal cellular features of maize ligule outgrowth. As a genetic model for de-novo organ axis establishment, the properties and possible mechanisms described in this manuscript are of great importance. This work is a thoughtful and pleasing set of observations and correlations between cellular properties and morphological phenomena. This manuscript is particularly valuable to workers in this field because the authors establish a system of morphological, molecular developmental stages pre- and post- ligule outgrowth. Although this work is descriptive, the authors provide thoughtful speculation and describe several fruitful experiments which would evaluate the necessity/sufficiency of the cellular phenomena observed for future workers.

The major findings of the manuscript are: 1) ligule anatomical stages can be identified based on a correlation with sheath length, 2) cell division and expansion patterns correlate with ligule developmental stages as well as their position relative to the ligule, 3) mechanical properties of cell walls vary over the developing leaf compartments and these differences change as the ligule begins to grow outwards, 4) PIN polarization is dynamic during ligule outgrowth, but non-polar in the ligule, 5) transcriptional markers of auxin signaling are low in early pre-ligule stages, but become detectable in late ligule outgrowth. The manuscript is well written and a pleasure to read. This type of article would be especially useful for the functional training of students or new workers in the leaf development field.

Comments for the author

I have a few minor requests that might improve the clarity for readers outside of this area of specialty. The sheath length staging system presented is a fantastic tool for workers in this field, it would help to understand how the described stages correlate with V2 or V3 leaf plastochron numbers which have been the basis for other work in this field. How long do leaf primordia dwell in any of these stages? >1 plastochron? just a few hours? Calibration to the developmental and/or calendar clock would be valuable for new workers in this field.

In Figure 2F, the authors present their quantification of cell division types across developmental stages. Displaying this data as connected dots along a continuous sheath length axis is potentially problematic since the axis does not represent repeated observation of the same growing individuals. I recommend using stacked bar charts separated by the authors' nice stages as a grouping factor. It is unclear what statistical methods the authors have used to analyze the effect of stage on the different division types, but I recommend a MANOVA where all four classes of divisions are related to the stage factor and individual, followed by single-sided ANOVA for each factor to help demonstrate quantitate difference.

Figure 3 makes use of a proximal = 0, distal = 1 positional system. This is not as clear as the negative,0,positive positional axis system used in figure 4. It is unclear how the LR is defined, and Figure S3 does not clarify the cell shape and size features that were used to assign this axis.
In Figure 5, additionally summarizing average polarization based on the developmental categories established by the manuscript would make comparing stages much easier for outside readers.

The out-of-plane signal in Figure 6 is troubling. Although the authors have stated that quantifications are taken from regions without this haze, Figure 6 F-J have significant background beyond regions marked as veins. Providing the normalized in this display figure with the original images in a supplement might be more reassuring. Additionally (as in Figure 2 and 5), summarizing DR5 signal intensity by developmental stage, rather than sheath length would simplify the data for readers outside of maize leaf development and better reflect the characteristic images presented.

© 2023. Published by The Company of Biologists under the terms of the Creative Commons Attribution License (https://creativecommons.org/licenses/by/4.0/).

Reviewer 3

Advance summary and potential significance to field

The manuscript "The maize preligule band is subdivided into distinct domains with contrasting cellular properties prior to ligule outgrowth" by Neher et al, is an interesting story that adds to our existing understanding of ligule development. The most important contribution of the presented research is the novel AFM data which investigates the rigidity of cells across the developing ligule region specifically during ligule outgrowth.

Comments for the author

Overall, I think that this is an interesting manuscript worthy of publication. I do however, have the following comments to improve the manuscript and help the reader follow more easily:

- As analyses rely upon identifying morphological features of the preligule band (PLB) this study focusses on the differences that lead up to ligule outgrowth, but does not tell us about the patterning stages (i.e. how the ligule region is specified). It would be of interest to know if there are any cellular/ auxin signalling differences prior to the ability to define a PLB.
- Overall, the manuscript is difficult to follow and I recommend editing to help the story flow more, particularly in the introduction and discussion sections. For example introducing the importance of different boundaries earlier in the introduction will bring the reader to the key information for the manuscript more quickly as at the moment it starts out with a heavy focus on meristems and meristem-organ boundaries.
- The research is focused on spatial analyses of the PLB but it is difficult for the reader to orient themselves within context of the leaf and PLB domain without clear cartoons/ representative images in each figure. Addition of these type of cartoons, with colour coding for the different domains/ subdomains to each figure would really help with clarity.
- It is not clear where in the medial-lateral, and sometimes proximal-distal axis the data is collected for the experiments.
- For each analysis it is not clear how cells are selected for analysis or where they are in the ligular region relative to each other. Please provide more information.
- Early PLB, PLB and late PLB are discussed as distinct phases of development with clear characteristics, but in Figure 2 PLB and Late PLB significantly overlap and the datapoints for each appear to be in the overlapping domain. If they overlap so significantly is it feasible to define them as different stages?
- Early PLB is not shown in Figure 1, why?
- As there are so many stages, it is important to make sure that the correct term is being used when discussing the data as this is confusing for the reader.
- In Figure 1 zoomed in images of the cells indicated in F would be very helpful- at the current resolution it is very difficult to see the differences.
- In Figure 2, inclusion of arrow heads/ color/ indicators in B/C/D and E would help readers who are not used to seeing images of the preprophase band.
- In Figure 3 the inclusions of a diagram or color-coded SEM image would help orient the reader with the locations of S/LR/PLR/DLR/B and would help clarify how the domains were defined.
- Figure 4 PLB is indicated as location 0 but the PLB is a region, the plots are hard to interpret relative to the spatial domains discussed. Also as the PLB at 0 is defined as having a relative IM of 1, it makes it confusing when the PLB in the text is described as having softer cells or more rigid cells in different domains and stages. For readers not used to AFM data the figure is not intuitive and it is hard to orient. Is there way to include diagrams/ SEM images/ annotations/ colour coding to help this?
- In Figure 6 fluorescent intensity is determined based on a set boxed area – were these normalised for cell number and/or size?
- As the authors mention DR5 is a late reporter of auxin response, have they looked at the DII venus reporter?
- The discussion focusses on pre-ligule and pre-auricle domains but the analysis is of the ligule before auricle differentiation?
- In the discussion the authors discuss the surface minimisation rule for divisions, are the authors proposing that an increase in cell depth drives ligule outgrowth as a mechanism?

© 2023. Published by The Company of Biologists under the terms of the Creative Commons Attribution License (https://creativecommons.org/licenses/by/4.0/).

- The main conclusion is that the ligule domain is progressively refined and subdomains are defined, this is a conclusion previously reported in both SEM and gene expression analyses. Perhaps modify this sentence (Line 516) to acknowledge that this is further evidence.

First revision

Author response to reviewers' comments

Dear Editor,

We were pleased to receive such detailed and constructive feedback on our submission. Most of their suggestions and concerns have been addressed, as outlined below.

Reviewer 1 Comments for the Author:

1. Figure 1 could be improved by providing:

a. Heat maps of cell sizes at different developmental stages; This could be also useful to better visualize the decrease in cell sizes observed during early boundary formation (Table S1). Authors have SEM and CSLM data, which should allow it to be easily generated.

b. Heat maps of surface curvature. This could help better distinguish early-stage PLB from late PLB.

The suggestion to create cell size and surface curvature heatmaps was very helpful. We used MorphoGraphX to generate these. We have made representative heatmaps for the early PLB through early fringe stages and they nicely supplement our other figures. We have added a new figure with the MorphoGraphX images, and incorporated MorphoGraphX analysis into Figure 4 (previously Fig. 3) as well.

2. Why the early PLB stage is not included in Figure 1?

We agree that including the early stage would be preferable. However, the early PLB stage is distinguishable by the increase in mitotic and pre-mitotic microtubule structures and PIN1 accumulation, so this stage is not easily visualized with SEM. We infer that enough new cross walls have not yet formed so it is difficult to see a recent division based solely on new cross walls in an SEM. We therefore unfortunately don't have any SEMs of this stage.

3. Lines 221-223: “These results show that spatially constrained divisions initially produce the ligule fringe, with cell expansion driving growth in the later stages.” – how do authors know that at early stages cellular growth is less important? It is possible that during the initiation of PLB, the growth is fast but is accompanied by a lot of divisions leading to a decrease in cell sizes.

We have modified the sentence. We did not intend to imply that the reduced size of the PLB cells must be due solely to different division rates, or that differences in expansion are unimportant at the early stages. The sentence now reads: *These results show that changes in division plane orientation contribute to early ligule development, with cell expansion driving elongation at the late fringe stage.*

4. Figure 3. It would be helpful to include representative confocal images (optical sections) of PLB at different developmental stages to visualize the thickening of the epidermis (similar to what is shown in the last figure). Representative images of the cell divisions should also be included.

(Now Figure 4) Thank you for the suggestion that an addtional visual aid would be helpful in this figure. Rather than confocal orthoslices, we added representative MorphoGraphX cell depth heatmaps generated from the confocal stacks to this figure, as well as an orthoslice highlighting a recent periclinal division, and a projection of the preligule ridge.

© 2023. Published by The Company of Biologists under the terms of the Creative Commons Attribution License (https://creativecommons.org/licenses/by/4.0/).

The addition of panel A functionally replaces former figure S3, outlining how the zones of the leaf were determined.

5. Lines 241-243: "Confocal projections suggested that differential thickening likely also occurs in underlying cell layers; however, reduced signal prevented measuring cell thickness in deeper layers." If mentioned, then please show the images.

Our confocal orthoslices imply this, but the signal in deeper cell layers is low and the stacks do not go deep enough to measure the depth of the underlying cells. Since this is not integral to the paper, we have removed the sentence.

6. Figure 3: Statistically significant differences should be indicated.

(Now Figure 4) We performed one-sample chi-square tests for variance, to test whether the distribution of each division type is uniform or not. Significant differences are now indicated in the figure. Figure legend and methods have been updated.

7. Lines 269-278: Most of this text should be moved to the method section. It breaks the flow of the story.
We agree, we revised this text to reduce the explanation of AFM and IM in order to maintain narrative flow, moving some of that text to the methods.

8. I am a bit surprised that the PLB regions seem to be the stiffest at the early stages. This is the region where cell divisions are the most intensive. It would be great to correlate the mechanical properties measured with AFM with the cellular growth rates at different developmental stages during ligule initiation.

Yes, one would expect new cell plates to be relatively weak/soft. We often see what we believe to be "young" anticlinal cell walls in the blade and sheath, which appear to be thinner. These putative more recent divisions are soler than the others. Possibly, the boundary function in the PLB may cause the new cell walls to stiffen more rapidly.

We have not yet explored time lapse imaging as a means of quantifying growth rates, although this would be an excellent experiment to perform in the future. We expect this experiment to be challenging because the leaf must be removed from the meristem prior to live imaging.

9. Lines 445-449: "The dramatic softening of cell walls in the proximal PLB preceding ligule outgrowth is highly reminiscent of data from the Arabidopsis SAM, where biochemical changes and mechanical softening in the cell walls of the subepidermal cell layers precede the outgrowth of leaf primordia (Peaucelle et al., 2011)." – I am not sure if the softening of the epidermal layer can be compared with subepidermal softening and between such distinct systems. Also, the softening of internal layers in the SAM was reported in incipient primordia – authors do not observe softening preceding PLB formation.

While softening does not precede PLB formation, nor the formation of the ridge at the PLB, it does precede the outgrowth of the ligule fringe from the ridge. We agree that the systems are not necessarily comparable, particularly given that the epidermis is probably under tension while deeper layers are under compression, but broadly, AFM often shows a decrease in elastic modulus to be associated with increased growth. We have therefore revised the sentence to be more general and to also reference data from the hypocotyl epidermis: *The dramatic softening of cell walls in the proximal PLB preceding ligule outgrowth is highly reminiscent of AFM experiments in Arabidopsis, where biochemical changes and mechanical softening in the cell walls correlate with increased growth (Peaucelle et al., 2011, Bou Daher et al., 2018).*

Reviewer 2 Comments for the Author:

I have a few minor requests that might improve the clarity for readers outside of this area of specialty. The sheath length staging system presented is a fantastic tool for workers in this field, it would help to understand how the described stages correlate with V2 or V3 leaf plastochron

© 2023. Published by The Company of Biologists under the terms of the Creative Commons Attribution License (https://creativecommons.org/licenses/by/4.0/).

numbers which have been the basis for other work in this field. How long do leaf primordia dwell in any of these stages? >1 plastochron? just a few hours? Calibration to the developmental and/or calendar clock would be valuable for new workers in this field.

The classic staging “V” staging was established using field grown maize, which is not directly comparable to our greenhouse growth conditions. It also counts the visible ligular region (also called collar) when it emerges from the furl, whereas all of the stages of ligule growth are occurring deep within the furl. For plastochron comparison, we can roughly match our early PLB, PLB, late PLB, and early fringe stages to plastochron numbers, which is temporal measure of the posi6on of a primordium since its emergence at the SAM. Again, the exact plastochron is difficult to determine when dissecting, but we can infer the plastochron when comparing our sheath height measurement with that in longitudinal sections as from Johnston et al. 2014. We therefore can identify our leaf stages by comparing sheath heights to get a comparable es6mate of plastochron number. We edited the text accordingly:

The early PLB stage, which was observed at sheath lengths of 0.3 – 1.1 mm, comparable to plastochron 6 (Johnston et al., 2014).

1.2 mm, comparable to late plastochron 6 and early plastochron 7 (Johnston et al., 2014)

1.9 mm, comparable to late plastochron 7 (Johnston et al., 2014)

Leaves in the early fringe stage had a median sheath length of 3.5 mm, comparable to plastochron 8.

In Figure 2F, the authors present their quatification of cell division types across developmental stages. Displaying this data as connected dots along a continuous sheath length axis is potentially problematic since the axis does not represent repeated observation of the same growing individuals. I recommend using stacked bar charts separated by the authors' nice stages as a grouping factor. It is unclear what statistical methods the authors have used to analyze the effect of stage on the different division types, but I recommend a MANOVA where all four classes of divisions are related to the stage factor and individual, followed by single-sided ANOVA for each factor to help demonstrate quantitate difference.

Thank you for these recommendations. We agree that the line graph could be interpreted to mean that a single sample is changing over time, a stacked bar graph is presented instead.

We have included the p-value from the MANOVA in the graph, with significance groups from the post-hoc of the single-sided ANOVAs added to Table S1. We tested for differences in the % of divisions between different orientations at each stage, and for differences in the % of each division orientation between stages.

Figure 3 makes use of a proximal = 0, distal = 1 posi6onal system. This is not as clear as the negative,0,positive positional axis system used in figure 4. It is unclear how the LR is defined, and Figure S3 does not clarify the cell shape and size features that were used to assign this axis.

(Now figure 4) We have included additional graphics, including a heatmap of cell size, that helps clearly outline how positions 0 and 1, and the zones of the ligular region were determined on the basis of cell size and shape. We also added a representative confocal of a periclinally-divided cell in the proximal ligular region, and cell depth heatmaps as additional visual aids.

In Figure 5, additionally summarizing average polarization based on the developmental categories established by the manuscript would make comparing stages much easier for outside readers.

(Now Figure 6) 6M shows average fluorescence intensity ratios (polarity) across developmental stages. We did not group the samples by stage, but we did modify the figure to more clearly delimit the stages relative to sheath length. We used the sheath length ranges from figure 1 to

© 2023. Published by The Company of Biologists under the terms of the Creative Commons Attribution License (https://creativecommons.org/licenses/by/4.0/).

outline the progression of developmental stages. However, PIN1 localization is not very polarized at any stage.

The out-of-plane signal in Figure 6 is troubling. Although the authors have stated that quantifications are taken from regions without this haze, Figure 6 F-J have significant background beyond regions marked as veins. Providing the normalized in this display figure with the original images in a supplement might be more reassuring. Additionally (as in Figure 2 and 5), summarizing DR5 signal intensity by developmental stage, rather than sheath length would simplify the data for readers outside of maize leaf development and better reflect the characteristic images presented.

(Now Figure 7) We now show normalized, background subtracted images for Figure 7F-J. As in the previous figure, we modified panel P to more clearly delimit the stages relative to sheath length, although signal is quite low at all stages.

Reviewer 3 Advance Summary and Potential Significance to Field:
Reviewer 3 Comments for the Author:

Overall, I think that this is an interesting manuscript worthy of publication. I do however, have the following comments to improve the manuscript and help the reader follow more easily:

-As analyses rely upon identifying morphological features of the preligule band (PLB) this study focusses on the differences that lead up to ligule outgrowth, but does not tell us about the patterning stages (i.e. how the ligule region is specified). It would be of interest to know if there are any cellular/ auxin signalling differences prior to the ability to define a PLB.

We agree that this is an ongoing interest and is an essential question. Currently, we have not detected any visible differences prior to the appearance of the PLB. However, prior transcriptomic experiments give some clues with regard to auxin responses in the early leaf. We have added to following text to the introduction:

Transcriptomic experiments indicate that prior to and during ligule development, auxin responses are higher in the blade than in the sheath (Leiboff et al., 2020, Johnston et al., 2014).

-Overall, the manuscript is difficult to follow and I recommend editing to help the story flow more, particularly in the introduction and discussion sections. For example introducing the importance of different boundaries earlier in the introduction will bring the reader to the keyinformation for the manuscript more quickly as at the moment it starts out with a heavy focus on meristems and meristem-organ boundaries.

We have edited the introduction to more clearly focus on boundaries and the PLB/ligule and removed discussion of leaf initiation in Arabidopsis. We agree that the introduction flows better by talking about boundaries earlier. We also made edits to the portions of the intro and discussion regarding auxin, with the aim of improving flow. Overall, the manuscript has been edited for clarity.

-The research is focused on spatial analyses of the PLB but it is difficult for the reader to orient themselves within context of the leaf and PLB domain without clear cartoons/ representative images in each figure. Addition of these type of cartoons, with colour coding for the different domains/ subdomains to each figure would really help with clarity.

Thanks for this suggestin. We have added leaf cartoons to figures 1 and 5 to help orient the reader. We also added arrows indicating the orientation of the proximal-distal axis in figures 1, 2, 3 and 4.

-It is not clear where in the medial-lateral, and sometimes proximal-distal axis the data is collected for the experiments.

© 2023. Published by The Company of Biologists under the terms of the Creative Commons Attribution License (https://creativecommons.org/licenses/by/4.0/).

The leaf cartoons help with this, as we outline the region of interest. Leaf regions are more clearly outlined for the cell depth and periclinal division figure. Leaf regions are more clearly outlined in the AFM sliding widow figure. We also added the following text to the methods: *Although the mediolateral position was not strictly controlled, images and measurements were collected from the lateral and marginal domains of the leaf primordium, not the central domain (Hay and Hake, 2004).*

-For each analysis it is not clear how cells are selected for analysis or where they are in the ligular region rela6ve to each other. Please provide more informa6on.

We agree that this could be more clear, par6cularly in what is now Figure 4. Therefore, we added a projection of a confocal scan outlining the zones of the leaf. We also added a supplemental movie outlining how periclinal divisions were identified.

With regard to our manual resampling of the AFM scans (Fig. S3) we added the following text to the methods:

We thoroughly sampled each cell in each proximodistal tissue region, resampling at least 5 indentations per wall category per cell.

With regard to the calculation of division orientation % in the ligular region, we considered all dividing cells in our images of the ligular region, but not in the blade or sheath. We have added the following text to the methods:

All imaged cells in the ligular region that had visible YFP-labelled preprophase bands or phragmoplasts were considered.

-Early PLB, PLB and late PLB are discussed as distinct phases of development with clear characteristis, but in Figure 2 PLB and Late PLB significantly overlap and the datapoints for each appear to be in the overlapping domain. If they overlap so significantly is it feasible to define them as different stages?

Leaf development is a progressive process, so while we present sheath length as a proxy for developmental stage and differences are statistically significant, sheath length is not an absolute predictor of exact leaf stage. Therefore, sheath length measurements overlap between PLB and Late PLB stages. Topography, cell size, frequency of periclinal divisions differ, and par6cularly the mechanical profile in the ligular region is distinctive between PLB and late PLB.

-Early PLB is not shown in Figure 1, why?

The early PLB is distinguishable only by the increase in mitotic and pre-mitotic microtubule structures, and PIN1 accumulation (as shown in Fig 6A) but the divisions have not happened yet so they are not easily seen with SEM.

-As there are so many stages, it is important to make sure that the correct term is being used when discussing the data as this is confusing for the reader.

We edited the text for clarity and made sure that the wording in the text is consistent with the terminology in the figures.

-In Figure 1 zoomed in images of the cells indicated in F would be very helpful- at the current resolution it is very difficult to see the differences.

Due to the low magnifica6on/resolution, zoomed in images were very grainy and did not help visualize the cells very well. We already have decent images of preprophase bands in figure 2, so we reduced Figure 1's focus on mitotic structures and eliminated the arrows/arrowheads, as they are not essential to understand this figure.

-In Figure 2, inclusion of arrow heads/ color/ indicators in B/C/D and E would help readers who

© 2023. Published by The Company of Biologists under the terms of the Creative Commons Attribution License (https://creativecommons.org/licenses/by/4.0/).

are not used to seeing images of the preprophase band.

We have revised this figure to more clearly indicate the preprophase band in different division planes. Cartoons now more clearly indicate both the 3D orientation of the PPBs and a 2D representation of what the PPB looks like in a single Z-slice, for easy comparison with the confocal cell images.

-In Figure 3 the inclusions of a diagram or color-coded SEM image would help orient the reader with the locations of S/LR/PLR/DLR/B and would help clarify how the domains were defined.

(Now Figure 4) We have included additional graphics to help orient the reader and to outline the differences between the sheath, ligular region, and blade to clarify how the zones, and position 0/1 were defined.

-Figure 4 PLB is indicated as location 0 but the PLB is a region, the plots are hard to interpret relative to the spatial domains discussed. Also as the PLB at 0 is defined as having a relative IM of 1, it makes it confusing when the PLB in the text is described as having softer cells or more rigid cells in different domains and stages. For readers not used to AFM data the figure is not intuitive and it is hard to orient. Is there way to include diagrams/ SEM images/ annotations/ colour coding to help this?

(Now Figure 5) We have added text to better explain this, and modified the figure. A blue box represents the sliding window, while red lines indicate the limits of the ligular region. This helps the reader see that the early mechanical pattern has a maximum in IM within the ligular region, while the late mechanical pattern has both a maximum and minimum in IM within the ligular region.

-In Figure 6 fluorescent intensity is determined based on a set boxed area – were these normalised for cell number and/or size?

(Now Figure 7) This were not normalized because the goal of this experiment was to evaluate the average DR5 response across a large area containing many cells. Normalization for cell size is not likely to add value, and furthermore we do not know of a good way to do this normalization. We did, however, do a background subtraction in the revised figure to eliminate the significant background signal.

-As the authors mention DR5 is a late reporter of auxin response, have they looked at the DII venus reporter?

We have not examined DII Venus for this study.

-The discussion focusses on pre-ligule and pre-auricle domains but the analysis is of the ligule before auricle differentiation?

We used the term pre-auricle because the cells in that position must at least contribute to the auricle, even if they have not yet become distinct. However, the distal PLB / preauricle cells on the adaxial side of the region are already distinct in terms of cell size and shape, relative to the incipient ligule and blade. We have not studied how the auricle develops in the ad to ab polarity of the leaf.

-In the discussion the authors discuss the surface minimisation rule for divisions, are the authors proposing that an increase in cell depth drives ligule outgrowth as a mechanism?

Although our data don't prove an increase in cell depth drives ligule outgrowth, we propose that an increase in cell depth creates a columnar cell geometry that particularly favors periclinal divisions, which are thought to be essential in the process.

-The main conclusion is that the ligule domain is progressively refined and subdomains are defined, this is a conclusion previously reported in both SEM and gene expression analyses. Perhaps modify this sentence (Line 516) to acknowledge that this is further evidence.

© 2023. Published by The Company of Biologists under the terms of the Creative Commons Attribution License (https://creativecommons.org/licenses/by/4.0/).

We have modified this sentence: *Our data support a model in which the boundary between blade and sheath in the maize leaf is progressively refined in the ligular region, producing two subdomains, as previously proposed based on SEM and gene expression data (Sylvester et al., 1990; Johnston et al., 2014).*

Second decision letter

MS ID#: DEVELOP/2023/201608

MS TITLE: The maize preligule band is subdivided into distinct domains with contrasting cellular properties prior to ligule outgrowth

AUTHORS: Wesley Neher, Carolyn G. Rasmussen, Siobhan A Braybrook, Vladimir Lazetic, Claire E. Stowers, Paul T. Mooney, Anne W. Sylvester, and Patricia S. Springer
ARTICLE TYPE: Research Article

I am happy to tell you that your manuscript has been accepted for publication in Development, pending our standard ethics checks. The reviewers had no additional suggestions and I agree with them that the revised manuscript is exciting, accessible and a wonderful addition to the plant developmental biology field.

Reviewer 1

Advance summary and potential significance to field

This manuscript brings new insights into the mechanism of maize ligule development. It precisely characterizes preligule band formation including the quantifications of cell geometry, division orientation, and cell wall mechanics underlying this process. Significantly it demonstrates that the initiation of this boundary is associated with the establishment of mechanically distinct epidermal domains.

Comments for the author

Thanks for addressing most of my comments and adding suggested qualifications. I am fully satisfied with these revisions and believe that your manuscript is not ready for publication.

Reviewer 2

Advance summary and potential significance to field

The authors have presented an improved version of their already exciting manuscript. Their thoughtful incorporation of reviewer requests have improved the clarity and potential reach of this work. Careful consideration of the anatomical, biophysical, and auxin hormone biology immediately preceding and leading into ligule formation will serve as an exciting comparator for lateral growth events more generally.

Comments for the author

The authors have addressed all issues provided by the last set of reviews. The current form of the manuscript is clear and exciting. I have no further requests or comments.

© 2023. Published by The Company of Biologists under the terms of the Creative Commons Attribution License (https://creativecommons.org/licenses/by/4.0/).

Reviewer 3

Advance summary and potential significance to field

I thank the authors for their consideration and detailed responses to all reviewer comments.

Comments for the author

I have no further suggested revisions.

© 2023. Published by The Company of Biologists under the terms of the Creative Commons Attribution License (https://creativecommons.org/licenses/by/4.0/).
